# Reprogramming of Mitochondrial Respiratory Chain Complex by Targeting SIRT3‐COX4I2 Axis Attenuates Osteoarthritis Progression

**DOI:** 10.1002/advs.202206144

**Published:** 2023-01-22

**Authors:** Yijian Zhang, Yang Liu, Mingzhuang Hou, Xiaowei Xia, Junlin Liu, Yong Xu, Qin Shi, Zhongmin Zhang, Liang Wang, Yifan Shen, Huilin Yang, Fan He, Xuesong Zhu

**Affiliations:** ^1^ Department of Orthopaedics The First Affiliated Hospital of Soochow University Soochow University Suzhou 215006 China; ^2^ Orthopaedic Institute Medical College Soochow University Suzhou 215007 China; ^3^ Department of Orthopedics Nanfang Hospital Southern Medical University Guangzhou 510515 China; ^4^ Department of Orthopedics The Third Affiliated Hospital Southern Medical University Guangzhou 510630 China; ^5^ Department of Orthopedic Surgery Zhejiang University School of Medicine Hangzhou 310003 China

**Keywords:** COX4I2, deacetylation, mitochondria, osteoarthritis, SIRT3

## Abstract

Mitochondrial homeostasis is of great importance for cartilage integrity and associated with the progression of osteoarthritis (OA); however, the underlying mechanisms are unknown. This study aims to investigate the role of mitochondrial deacetylation reaction and investigate the mechanistic relationship OA development. Silent mating type information regulation 2 homolog 3 (SIRT3) expression has a negative correlation with the severity of OA in both human arthritic cartilage and mice inflammatory chondrocytes. Global SIRT3 deletion accelerates pathological phenotype in post‐traumatic OA mice, as evidenced by cartilage extracellular matrix collapse, osteophyte formation, and synovial macrophage M1 polarization. Mechanistically, SIRT3 prevents OA progression by targeting and deacetylating cytochrome c oxidase subunit 4 isoform 2 (COX4I2) to maintain mitochondrial homeostasis at the post‐translational level. The activation of SIRT3 by honokiol restores cartilage metabolic equilibrium and protects mice from the development of post‐traumatic OA. Collectively, the loss of mitochondrial SIRT3 is essential for the development of OA, whereas SIRT3‐mediated proteins deacetylation of COX4I2 rescues OA‐impaired mitochondrial respiratory chain functions to improve the OA phenotype. Herein, the induction of SIRT3 provides a novel therapeutic candidate for OA treatment.

## Graphical abstract

The SIRT3‐medicated post‐translational acetylation of the mitochondrial respiratory chain has therapeutic potential for the treatment of osteoarthritis (OA). Mechanistically, SIRT3 directly binds and deacetylates COX4I2 to promote mitochondrial respiration activity. Meanwhile, SIRT3‐mediated deacetylation of SOD2 reinforces mitochondrial intrinsic antioxidant properties. Synergistically, strengthening mitochondria by SIRT3 ensures extracellular matrix anabolism and catabolism dynamic balance to ultimately achieve OA amelioration.

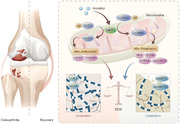

## 1 Introduction

Osteoarthritis (OA), the common form of arthritis, is rapidly becoming the most intractable chronic human health disorder, with a high socioeconomic cost and the leading cause of disability. It is estimated that OA affects over 500 million (7%) people globally.^[^ ^1^ ^]^ Worth noting, that the prevalence of adults over 50 years is as high as 50%, with serious impacts on quality of life.^[^ ^2^ ^]^ However, current therapeutic options for OA such as analgesics, nonsteroidal anti‐inflammatory agents, or physical treatment are not favorable, especially for end‐stage patients. Cartilage erosion, subchondral bone sclerosis, and synovial inflammation, collectively known as the “troika” in the pathogenic process, all contribute to the development of OA. Among the three pathological features, cartilage degeneration caused by chondrocyte apoptosis or extracellular matrix (ECM) degradation is the most common and is responsible for overall joint arthropathy. Over‐activation of proteinases like matrix metalloproteinases (MMPs) or a disintegrin and metalloproteinase with thrombospondin motifs (ADAMTS), in an arthritic environment can destroy intact ECM architectures composed of collagens and proteoglycans.^[^ ^3^ ^]^ Therefore, new targets to break the vicious circle of ECM loss and OA development are required.

Mitochondrion is organelle found in large numbers in most cells including chondrocytes, where the biochemical processes of aerobic respiration and energy production occur.^[^ ^4^ ^]^ Mitochondrial dysfunction has been associated with multiple musculoskeletal diseases including osteoporosis, intervertebral disc degeneration, or sarcopenia.^[^ ^5^ ^]^ Sigma‐1 receptor, a chaperone located on the mitochondria‐associated endoplasmic reticulum membrane, has been identified as a negative regulator of osteoclastogenesis and may be a candidate for osteoporosis treatment.^[^ ^6^ ^]^ In avascular and low‐oxygen environments, articular chondrocytes generally survive and maintain integrity. However, damaged mitochondria‐initiated energy depletion, free radicals’ accumulation, mitochondrial DNA (mtDNA) mutations, or calcium overload synergistically trigger and accelerate the progression of OA.^[^ ^7^ ^]^ Our recent research showed that OA chondrocytes have mitochondrial dysfunction and oxidative stress disorders. During OA pathogenesis, melatonin‐mediated mitochondrial recharge protects cartilage from ECM degradation.^[^ ^8^ ^]^ Similarly, Zhang et al. prepared a camouflaged meta‐defensome to transform M1 synovial macrophages into the M2 phenotype, allowing mitochondrial metabolic reprogramming for antagonizing OA.^[^ ^9^ ^]^ However, the specific mechanisms by which mitochondria affect OA as well as the pivotal regulators involved are still largely unknown.

Silent mating type information regulation 2 homolog 3 (SIRT3), a sirtuin family member, is preferentially localized to mitochondria where it functions as deacetylase enzymes.^[^ ^10^ ^]^ The SIRT3 positively regulates the activities of target proteins in mitochondria dependently in a post‐transcript manner via deacetylation of lysine residues. The SIRT3 deacetylases and activates the function of mitochondrial manganese superoxide dismutase (SOD2) to scavenge the over‐generation of reactive oxygen species (ROS).^[^ ^11^ ^]^ Additionally, SIRT3‐SOD2 signaling pathway enhancement protects cartilage from oxidative stress injury.^[^ ^12^ ^]^ Beyond maintenance of redox balance, SIRT3 enhances energy metabolism by directly deacetylating and animating mitochondrial isocitrate dehydrogenase 2,^[^ ^13^ ^]^ a key substrate in the tricarboxylic acid (TCA) cycle. As the downstream workshop of TCA, mitochondrial respiratory chain (MRC) is in charge of energy production via catalyzing oxidation, transferring the electrons, and generating the adenosine triphosphate (ATP). The complex IV cytochrome‐c oxidase (COX) serves as the terminal enzyme of MRC, transferring the electron from reduced cytochrome c to oxygen, and hence allowing ATP production.^[^ ^14^ ^]^ Importantly, cytochrome c oxidase subunit 4 isoform 2 (*Cox4i2* ^−/−^) deletion impairs mitochondrial membrane potential (*ΔΨm*) polarization and compromises electron transport chain (ETC).^[^ ^15^ ^]^ However, the exact roles of SIRT3 and MRC in the development of OA have not as yet been established.

In this study, SIRT3 global knockout mice (*Sirt3* ^−/−^) were constructed using the CRISPR/Cas9 system to investigate the key action of mitochondrial SIRT3 on protecting OA and the underlying mechanisms involved. Specifically, proteomics sequence combined with protein interaction assays revealed that SIRT3 regulates COX4I2 in a deacetylation‐dependent manner. Furthermore, SIRT3 natural agonist honokiol was intra‐articularly injected into post‐traumatic OA mice to determine the effect of SIRT3 activation on rescuing cartilage degeneration in vivo.

## 2 Results

### 2.1 Development of OA Was Associated with Mitochondrial SIRT3 Loss

**Figure 1:**
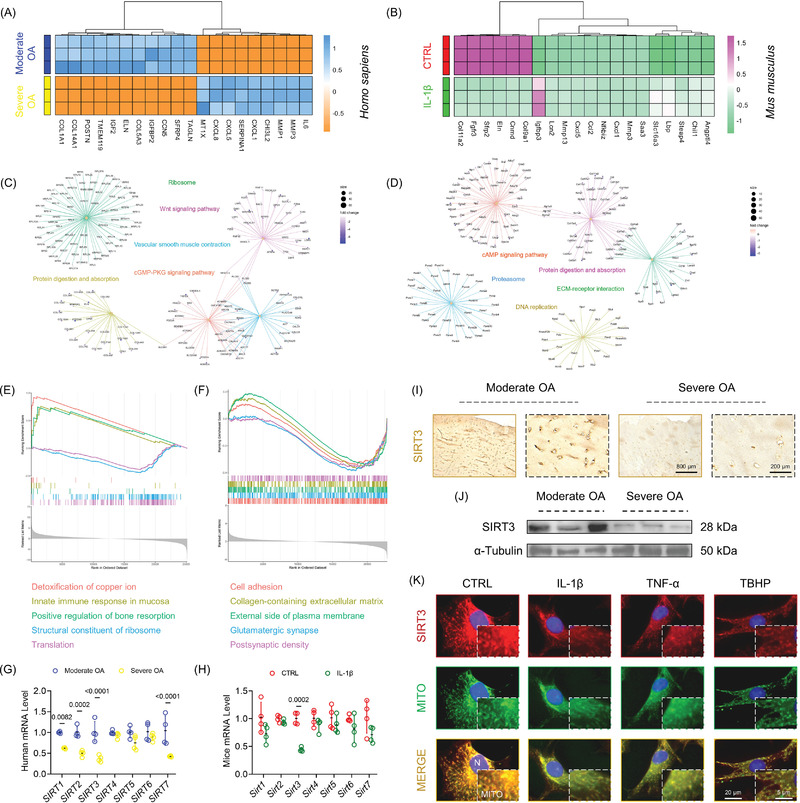
Cartilage ECM balance and mitochondrial SIRT3 expression were correlated with the progression of OA. A,B) Heatmaps of the top 20 differentially expressed genes in human or mice OA cartilage samples. C,D) Biological enrichments based on Kyoto Encyclopedia of Genes and Genomes (KEGG) database resources. E,F) Biological enrichments based on Gene Ontology (GO) database resources. G,H) Transcript levels of the sirtuins family (SIRT1‐7) were determined with RT‐PCR assays. I,J) The expression of SIRT3 in human OA cartilage was determined using immunostaining or immunoblotting. K) The expression of SIRT3 in proinflammatory cytokine‐treated mice chondrocytes was measured using immunofluorescence staining. Data are presented as mean ± SD of at least three independent assays for each experiment. Statistically significant differences between groups are set at *p* <0.05.

To investigate the pathological changes in OA, moderately or severely injured articular cartilage was collected from knee arthroplasty patients. In severe OA cases, the radiological images showed torn cartilage and narrowed joint space (Figure S1A, Supporting Information). A lower clinical score on the K‐L grades or Outerbridge scale predicted poorer knee joint functions (Figure S1B, Supporting Information). Histological analysis revealed decreased staining with acidic proteoglycans in cartilage tissues, as well as elevated Osteoarthritis Research Society International (OARSI) scores (Figure S1C, Supporting Information). Moreover, the metabolic balance of cartilage ECM was completely disrupted in severe arthritic chondrocytes (Figure S1D,E, Supporting Information). Subsequently, murine‐derived chondrocytes were treated with three recognized proinflammatory cytokines interleukin (IL)‐1*β*, tumor necrosis factor (TNF)‐*α*, and *tert*‐butyl hydroperoxide (TBHP), respectively, to comprehensively simulate an arthritic environment in vitro. Inflammation attacks destroyed matrix homeostasis, with IL‐1*β* exhibiting the optimal‐established effects (Figure S2A–D, Supporting Information). Subsequently, transcriptome sequencing was performed to reveal the underlying mechanisms involved in the OA development in human or murine cartilage (Figure S3A,B, Supporting Information). Imbalance of ECM metabolism, for example, decreased collagen fractions and increased proteinases abundance, was found with OA progression (**Figure** **1**A,B). Enrichment analysis indicated that protein modifications (post‐transcriptional and translation) and energy metabolism (cAMP signaling and ATP biosynthetic process) were highly correlated (Figure 1C–F and Figure S3C, Supporting Information). Given the critical roles of sirtuins‐mediated epigenetic alteration and energy regulation in mammals, the expression of sirtuins family (SIRT1‐7) was detected, in which mitochondrial SIRT3 was altered impressively at the mRNA level (Figure 1G,H). Correspondingly, SIRT3 protein expression in human cartilage samples was downregulated, both in tissue and cellular levels (Figure 1I,J and Figure S3D, Supporting Information). Mice chondrocytes treated with proinflammatory cytokines also had a lower SIRT3 expression level (Figure 1K and Figure S3E, Supporting Information). The findings showed that ECM metabolic disturbance and mitochondrial SIRT3 loss were correlated with OA progression.

### 2.2 Deficiency of SIRT3 Exacerbated Cartilage Degeneration in OA Mice

**Figure 2:**
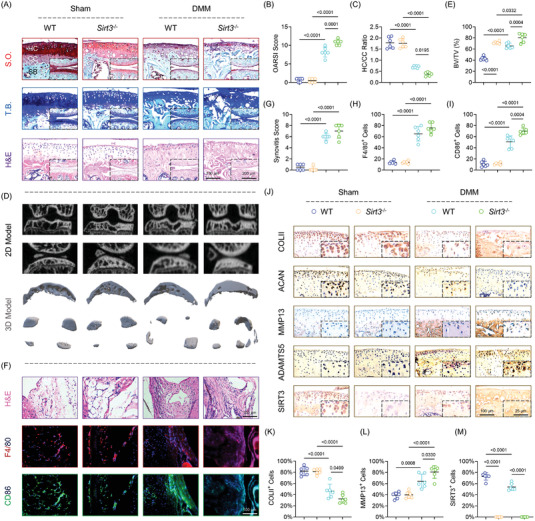
Global deletion of SIRT3 aggregated the progression of OA in post‐traumatic mice. A) Representative images of S.O. (top), hematoxylin and eosin (H&E, middle), and T.B. staining (bottom) in wild‐Type (WT) or *Sirt3^−/−^* mice at 8 weeks postoperatively (*n* = 6). B,C) Quantitative analysis of the OARSI scale or hyaline versus calcified cartilage (HC/CC) ratio (*n* = 6). D) Representative images of subchondral bone using micro‐computed tomography (µCT) reconstruction (*n* = 6). E) Quantitative analysis of the ratio of bone volume versus tissue volume (BV/TV). F) Representative images of H&E staining (top), immunofluorescence of F4/80 (middle), and CD86 (bottom) in the synovium (*n* = 6). G–I) Quantification of synovitis score, F4/80, or CD86‐positive macrophages (*n* = 6). J) Representative images of immunohistochemistry of COLII, ACAN, MMP13, ADAMTS5, and SIRT3 in articular cartilage (*n* = 6). K–M) Quantification of COLII, MMP13, or SIRT3‐positive chondrocytes. Data are presented as mean ± SD. Statistically significant differences between groups are set at *p*< 0.05.

Subsequently, we used the CRISPR/Cas9 system to create SIRT3 global knockout (*Sirt3^−/−^*) mice (Figure S4A,B, Supporting Information). Compared with wild‐type (WT) mice, SIRT3‐deficient mice have normal morphology (Figure S4C–E, Supporting Information). Unexpectedly, the axial skeleton development may be affected by SIRT3 loss, as manifested by shorter tail length (Figure S4F, Supporting Information). The WT or *Sirt3^−/−^* mice was subjected to post‐traumatic OA using classical destabilization of the medial meniscus (DMM) surgery to investigate the role of SIRT3 in OA development. The cartilage erosion was observed in DMM mice 8 weeks after surgery. Moreover, SIRT3 knockout mice aggregated the degeneration phenotype (**Figure** **2**A–C). After DMM surgery, subchondral bone sclerosis and bone formation increased as was expected. Specifically, knockout of SIRT3 promoted remodeling of subchondral bone in normal mice, indicating the negative impact on osteoblast and osteoclast recoupling (Figure 2D,E and Figure S4G,H, Supporting Information). Synovial proliferation and hypertrophy were exacerbated in *Sirt3^−/−^* mice with post‐traumatic OA according to histological staining (Figure 2F,G). In the absence of SIRT3, macrophage polarization balance was disrupted, as evidenced by over‐expressed F4/80, CD86 (M1 phenotype), and IL‐1*β* (inflammation marker), but stationary CD206 expression (M2 phenotype) (Figure 2H,I and Figure S4I,J, Supporting Information). Furthermore, *Sirt3^−/−^* OA mice developed uncoordinated ECM metabolism, as demonstrated by chaos in synthetic proteins (COLII and ACAN) and degrading enzymes (MMP13 and ADAMTS5) (Figure 2J–M and Figure S3K, Supporting Information). These findings revealed that global deletion of SIRT3 accelerated OA development in traumatic OA in vivo; more importantly, the pathogenesis involved the cross‐talk between articular cartilage, subchondral bone, and synovium.

### 2.3 Deletion of SIRT3 Impaired Chondrocytes’ Respiratory Functions

**Figure 3:**
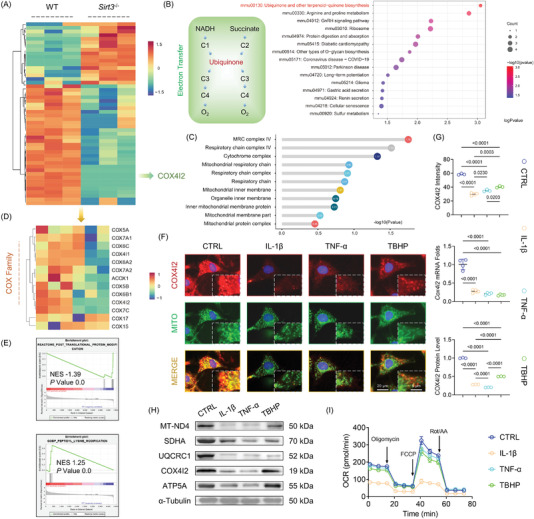
SIRT3 deficiency contributed to COX4I2 loss and MRC dysfunction. A) Heatmap indicated the differentially expressed proteins between WT or *Sirt3^−/−^* mice. B) KEGG enrichment analysis according to the differentially expressed proteins. C) The alteration of mitochondrial respiratory chain (MRC) functions and the corresponding *p* value. D) Heatmap indicated the differentially expressed MRC components between WT or *Sirt3^−/−^* mice. E) Gene set enrichment analysis (GSEA) of post‐translational protein modification and lysine modification. F,G) The expression of COX4I2 in proinflammatory cytokine‐treated mice chondrocytes were measured using immunostaining or immunoblotting. H) Protein levels of MT‐ND4, SDHA, UQCRC1, COX4I2, and ATP5A were measured using western blot. I) The oxygen consumption rate (OCR) was measured using a XF96 SeaHorse Analyzer. Data are presented as mean ± SD of at least three independent assays for each experiment. Statistically significant differences between groups are set at *p* < 0.05.

To elucidate the underlying mechanisms by which SIRT3 regulated cartilage homeostasis and affected OA progression, proteomic sequencing was performed using the articular chondrocytes from the WT or *Sirt3* ^−/^ *^−^* mice due to the post‐translational modifications by SIRT3. In response to SIRT3 knockout, 82 differentially expressed proteins were identified, with 17 proteins upregulated and 65 proteins downregulated (**Figure** **3**A and Figure S5A, Supporting Information). Gene Ontology analysis corroborated the impacts of SIRT3 deficiency on cartilage metabolism especially for the ECM organization and cartilage or bone development (Figure S5B, Supporting Information). Remarkably, considering the mitochondrial‐localized characteristics, MRC‐associated COX4I2 was selected as the hub protein. Meanwhile, Kyoto Encyclopedia of Genes and Genomes (KEGG) enrichment indicated the ubiquinone and the other terpenoid‐quinone biosynthesis pathway (mmu00130), which serves as the core in the reactions of electron transport and oxidative phosphorylation (OXPHOS) process at the mitochondria (Figure 3B). Further enrichment based on mitochondrial functions substantiated and confirmed the critical role of the MRC complex (Figure 3C). A heatmap was used to display the dysregulated level COX family by SIRT3 knockout in addition to the COX4I2 (Figure 3D). The gene set enrichment analysis revealed that SIRT3‐induced downstream signaling pathways may be dependent on post‐translational protein modification or lysine modification (Figure 3E). Subsequently, in vitro experiments revealed that proinflammatory stimuli reduced the expression of mitochondrial COX4I2 (Figure 3F,G). The corresponding molecular assays illustrated that deletion of SIRT3 completely disrupted the MRC complex superfamily, which included complex I–V (Figure 3H and Figure S5C,D, Supporting Information). Moreover, according to the seahorse assay, mitochondrial OXPHOS was negatively regulated by inflammatory stimuli, as evidenced by impaired basal respiration, ATP production, maximal respiration, and spare respiratory capacity (Figure 3I and Figure S5E, Supporting Information). These findings suggested that the absence of SIRT3 compromised the respiratory function of chondrocytes, primarily through the deterioration of MRC complex proteins.

### 2.4 SIRT3 Directly Deacetylates COX4I2 to Elicit MRC Complex Functions

**Figure 4:**
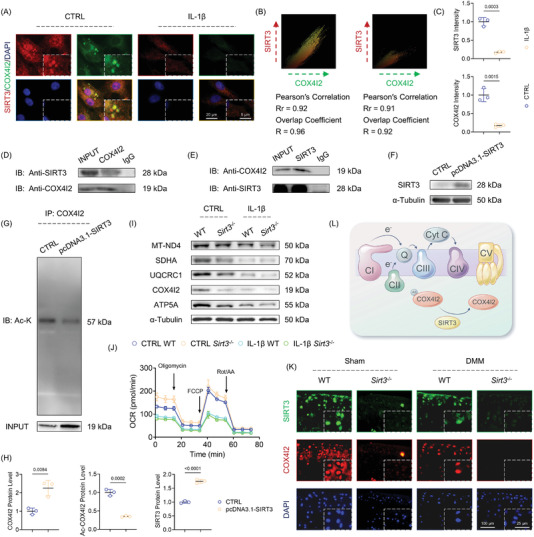
SIRT3 targeted and deacetylated COX4I2 in mitochondria in a post‐translational manner. A) The representative images of colocalization of SIRT3 and COX4I2 within mitochondria of CTRL or IL‐1*β*‐treated mice chondrocytes. B) Scattergrams indicated the fluorescence intensity and Pearson's correlation (Rr) and overlap correlation (*R*). C) Quantification of SIRT3 and COX4I2 immunofluorescence intensity. D,E) The interaction of SIRT3 and COX4I2 was validated using co‐immunoprecipitation CO‐IP assays. F,G) The acetylation level of COX4I2 was determined with immunoprecipitation. H) Quantitative protein levels of SIRT3, COX4I2, and acetylated COX4I2 (Ac‐COX4I2). I) Protein levels of ND4, SDHA, UQCRC1, COX4I2, and ATP5A were measured using western blot. J) The oxygen consumption rate (OCR) was measured using a XF96 SeaHorse Analyzer. K) The representative images of double fluorescent immunostaining for SIRT3 with COX4I2 in WT or *Sirt3^−/−^* mice at 8 weeks postoperatively (*n* = 6). L) Schematic diagram denoting the underlying mechanisms by which SIRT3 modulated MRC functions by targeting COX4I2. Data are presented as mean ± SD of at least three independent assays for each experiment. Statistically significant differences between groups are set at *p* <0.05.

To evaluate how SIRT3 affected the expression and function at the post‐translational level, chondrocytes were simultaneously co‐stained with SIRT3 and COX4I2. The colocalization of these two proteins within the mitochondria was demonstrated by the highly correlated Pearson's or overlap value in the fluorescence images (**Figure** **4**A–C). Forward and reverse co‐immunoprecipitation (Co‐IP) assays using chondrocyte extracts were used to validate the subcellular interaction between SIRT3 and COX4I2 (Figure 4D–F). Furthermore, the acetylation level of COX4I2 lysine by SIRT3 was determined using SIRT3‐mediated deacetylase activity. The immunoblotting assays revealed that SIRT3 not only promoted translational level of COX4I2, but reduced its acetylation level by deacetylation of specific lysine residues (Figure 4G,H). Then, chondrocytes derived from WT or *Sirt3^−/−^* mice were treated with IL‐1*β* in vitro to determine the indispensable role of SIRT3 in maintaining mitochondrial functions. The deletion of SIRT3 resulted in MRC complex defects at the mRNA and protein levels based on the findings (Figure 4I and Figure S6A,B, Supporting Information). Mitochondrial polarization and antioxidant properties were of impaired function in response to SIRT3 loss (Figure S6C–E, Supporting Information). Moreover, depletion of SIRT3 compromised MRC complex, subsequently leading to mitochondrial aerobic respiration suppression (Figure 4J and Figure S6G, Supporting Information). In vivo co‐staining corroborated the colocalization of SIRT3‐COX4I2. Moreover, the progression of OA accelerated the loss of COX4I2 in articular chondrocytes, and this phenotype was further exacerbated with knockout of SIRT3 (Figure 4K and Figure S6F, Supporting Information). These findings supported the hypothesis that SIRT3 preserved MRC complex functions by positively regulating COX4I2 activity through catalyzing deacetylation reactions (Figure 4L).

### 2.5 SIRT3 Activation by Honokiol Stabilizes ECM via Mitochondrial Enhancement

**Figure 5:**
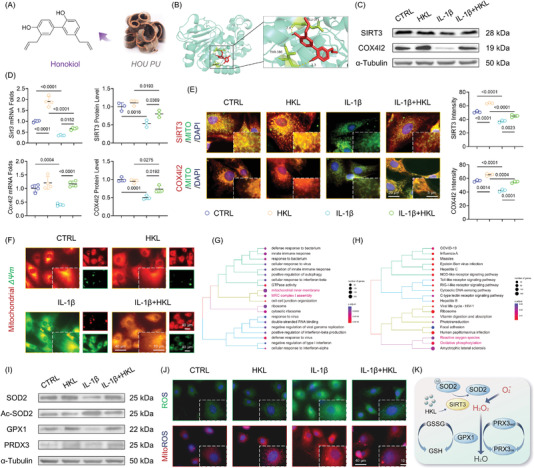
Activation of SIRT3 by honokiol advocated mitochondrial antioxidant defense to persevere cartilage ECM. A) The molecular structure of *HOUPU*‐derived honokiol. B) Molecular docking of honokiol‐SIRT3 complex. C,D) The transcript and translation level of SIRT3‐COX4I2 by the treatment of honokiol. E) The SIRT3 and COX4I2 expression within mitochondria was determined using fluorescent immunostaining. F) The mitochondrial membrane potential was monitored using the JC‐1 dye. G,H) Biological process and KEGG enrichment analysis based on differentially expressed genes after honokiol treatment. I) Protein levels of SOD2, Ac‐SOD2, GPX1, and PRDX3 were measured using western blot. J) Intracellular or mitochondrial ROS level after honokiol treatment. K) Schematic diagram representing molecular pathways of honokiol‐mediated SIRT3 activation protecting mitochondrial redox balance. Data are presented as mean ± SD of at least three independent assays for each experiment. Statistically significant differences between groups are set at *p* <0.05.

A small bioactive molecule honokiol extracted from *HOUPU* was identified as the SIRT3 agonist using a traditional Chinese medicine database search (**Figure** **5**A). Molecular docking revealed that honokiol bound to the SIRT3 protein at the GLU371 and THR380  with a binding energy of −4.45 kcal mol^−1^ (Figure 5B). The subsequent experiments corroborated the activation of honokiol on the SIRT3‐COX4I2 axis, particularly when treated with proinflammatory cytokines (Figure 5C–E). Honokiol treatment recovered mitochondrial functions in IL‐1*β*‐stimulated chondrocytes by activating SIRT3 (Figure 5F and Figure S7A, Supporting Information). The results of transcriptome sequencing confirmed that activation of SIRT3 by honokiol could modulate mitochondrial structure and function. Intriguingly, oxidative stress and ROS pathways were enriched by KEGG analysis, implying that HKL‐SIRT3 may affect mitochondrial ROS scavenging (Figure 5G,H and Figure S7F,G, Supporting Information). Based on this, the activities of mitochondria‐specific antioxidant elements such as SOD2, GPX1, or PRDX3 were fully evaluated. In IL‐1*β*‐stimulated chondrocytes, in vitro, honokiol treatment restored impaired mitochondrial antioxidant properties. The acetylation level of SOD2, a key free radical scavenger in mitochondria, was reduced by HKL‐activated SIRT3 (Figure 5I and Figure S7B,C, Supporting Information). Accordingly, the generation of free radicals was inhibited by honokiol treatment in the arthritic inflammatory environment (Figure 5J and Figure S7D, Supporting Information). Subsequently, honokiol treatment restored the disrupted anabolic or catabolic dynamic of murine chondrocytes in an arthritic environment (Figure S8A–C, Supporting Information). The results showed that honokiol‐activated SIRT3 recoupled cartilage ECM via mitochondrial reinforcement and enhancement of intrinsic mitochondrial antioxidant shield to inhibit mitochondrial ROS generation (Figure 5K).

### 2.6 Intra‐Articular Injection of Honokiol Ameliorated Surgically Induced OA Progression

**Figure 6:**
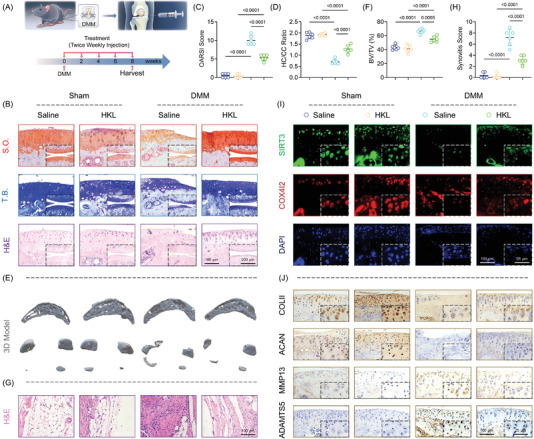
In vivo mobilization of SIRT3‐COX4I2 by honokiol prevented OA development. A) Schematic diagram representing the treatment strategy (*n* = 6). B) Representative images of S.O. (top), T.B. (middle), and H&E staining (bottom) at 8 weeks postoperatively (*n* = 6). C,D) Quantitative analysis of the OARSI scale or hyaline versus calcified cartilage (HC/CC) ratio (*n* = 6). E,F) Representative images of subchondral bone using micro‐computed tomography (µCT) reconstruction and quantitative analysis of the ratio of BV/TV (*n* = 6). G,H) Representative images of H&E staining in the synovium and quantitative analysis of the synovial inflammation (*n* = 6). I) Representative images of double fluorescent immunostaining for SIRT3 and COX4I2 at 8 weeks postoperatively (*n* = 6). J) Representative images of immunohistochemistry of COLII, ACAN, MMP13, and ADAMTS5 in articular cartilage (*n* = 6). Data are presented as mean ± SD. Statistically significant differences between groups were determined at a threshold of *p* <0.05.

**Figure 7:**
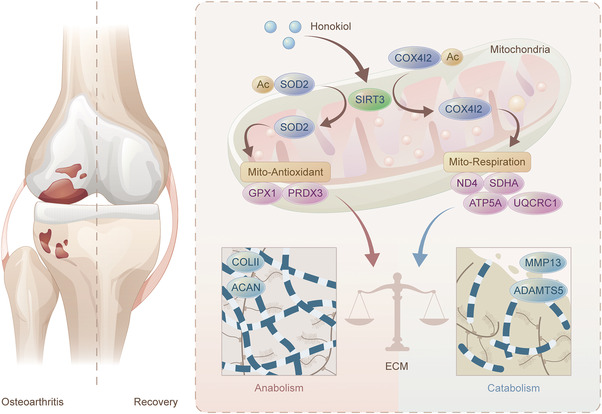
Targeting SIRT3 protects cartilage metabolic equilibrium through mitochondrial function retrieval. Mechanistically, SIRT3 directly binds and deacetylates COX4I2 to promote mitochondrial respiration activity. Meanwhile, SIRT3‐mediated deacetylation of SOD2 reinforces mitochondrial intrinsic antioxidant properties. Synergistically, strengthened mitochondria by SIRT3 ensure ECM anabolism and catabolism dynamic balance to ultimately achieve OA amelioration.

The DMM surgically induced mice were injected with honokiol intra‐articularly twice a week to assess the potential therapeutic effects of SIRT3 activation in preventing OA development (**Figure** **6**A). Honokiol treatment reduced cartilage destruction, as evidenced by decreased OARSI score and improved hyaline cartilage (HC) versus calcified cartilage (CC) ratio (Figure 6B–D). Furthermore, honokiol treatment alleviated the abnormal formation of osteophytes (Figure 6E,F and Figure S9A,B, Supporting Information) as well as overactivated synovial inflammation or M1 macrophage polarization (Figure 6G,H and Figure S9C,D, Supporting Information). The activation of the SIRT3‐COX4I2 axis in articular chondrocytes by honokiol treatment was confirmed by co‐staining results (Figure 6I and Figure S9E, Supporting Information). Importantly, in honokiol‐treated OA mice, collagen and proteoglycan expression was restored while matrix proteinases were suppressed, indicating that in vivo mobilization of SIRT3‐COX4I2 was able to remodel ECM homeostasis and impede OA progression (Figure 6J and Figure S9F, Supporting Information). The findings suggested that in situ injections of SIRT3 agonist honokiol attenuated traumatic OA development through intrinsic SIRT3‐COX4I2 activation (**Figure** **7**).

## 3 Discussion

Most life forms rely on mitochondria which are essential organelles for several pivotal cellular processes such as respiration and ATP synthesis. Because there are no blood or lymphatic vessels, or neural elements entering articular cartilage, traditional views suggest that anaerobic glycolysis dominates the fate of cartilage and modulates cartilage homeostasis.^[^ ^16^ ^]^ However, noticeable differences in the distribution of oxygen tension have been observed across the cartilage,^[^ ^17^ ^]^ with superficial hyaline chondrocytes receiving more oxygen tension than the CC in deepest regions receiving only extremely low oxygen tension.^[^ ^18^ ^]^ This is an evidence that aerobic respiration and ATP generation may determine the hyaline chondrocytes survival or self‐renewal. Human OA chondrocytes had impaired mitochondria biogenesis and respiratory chain functioning.^[^ ^19^ ^]^ Oligomycin‐mediated MRC dysfunction causes free radicals’ accumulation and chondrocyte death in an autophagy‐dependent manner.^[^ ^20^ ^]^ In developing cartilage, respiratory chain inactivation induced by the cartilage‐specific expression of an mtDNA helicase could disrupt the balance of aerobic or anaerobic respiration and cause a defect in energy production^[^ ^21^ ^]^ which further retardates ECM deposition and growth plate development.^[^ ^22^ ^]^ Chondrocytes’ apoptosis and depolarization of *ΔΨm* were initiated by nitric oxide‐induced inflammatory environment; the activity of cartilage MRC was compromised especially for complex IV (COX family).^[^ ^23^ ^]^ According to our findings, an isoform of COX4 subunits, COX4I2 is indispensable for the maintenance of cartilage integrity. Fukuda et al. found that hypoxia‐inducible factor‐1 regulates COX4 subunits’ expression by activating the transcript level of encoding COX4I2, hence manipulating COX family activities, ATP generation, and ROS production.^[^ ^24^ ^]^ Depletion of COX4I1 or COX4I2 causes a combined respiratory chain deficiency such as the missing of complex I, III, and IV assembly, as well as reduced respiration capacity or energy supply.^[^ ^25^ ^]^ Beyond complex IV, other MRC complexes are strongly implicated in the regulation of cartilage metabolism. Arthritic cartilage from OA patients showed decreased activities of MRC complex compared with the normal tissue.^[^ ^26^ ^]^ More specifically, deletion of NDUFS4, an essential complex I subunit, causes systemic inflammation and osteopetrosis by shifting osteoclasts to macrophages,^[^ ^27^ ^]^ both of which are capable of driving the progression of OA. Additionally, articular cartilage of the STR/ORT mice (spontaneous OA model) displays an early sign of metabolic dysfunction, as reflected by decreased lactate and dehydrogenase complex II activity.^[^ ^28^ ^]^ The potential mode of action of COX4I2 and the involvement of other MRC components will be investigated in our future research.

Metabolic regulation of OA epigenetics has received a lot of attention in recent years. Targeting DNA methyltransferase (DNMT) DNMT1/DNMT3a has therapeutic potential for treating OA via peroxisome proliferator‐activated receptor‐gamma (PPAR*γ*) promoter hypermethylation.^[^ ^29^ ^]^ In the presence of NAD^+^, the sirtuins family act as metabolic sensors to regulate cellular homeostasis and health.^[^ ^30^ ^]^ Originally discovered as a longevity protein in yeast, SIRT1 has been identified to mediate mammalian metabolic responses to calorie restriction or nutrient starvation.^[^ ^31^ ^]^ In surgically induced OA mice, cartilage‐specific deletion of SIRT1 accelerates ECM collapse and cartilage destruction.^[^ ^32^ ^]^ However, over‐expression of SIRT1 directly increases proinflammatory cytokine production of synovial cells, contributing to chronic inflammation in rheumatoid arthritis (RA). This suggests that SIRT1 reciprocally regulates OA or RA process.^[^ ^33^ ^]^ The SIRT6, a nuclear, chromatin‐associated protein, has been found to affect lifespan or aging in mammals through the coordination of insulin‐like growth factor (IGF)‐1 and IGF‐binding protein 1,^[^ ^34^ ^]^ or by promoting normal DNA repair.^[^ ^35^ ^]^ In human chondrocytes, SIRT6 deletion increases DNA damage, telomere dysfunction, and subsequent premature senescence.^[^ ^36^ ^]^ As another energy‐sensitive deacetylase protein, SIRT3 can modulate mitochondrial proteins by deacetylating lysine residues.^[^ ^37^ ^]^ More than half of basal ATP levels are lost in the heart, kidney, and liver of *Sirt3* ^−/^ *^−^* mice. Specifically, SIRT3 binds and deacetylases nicotinamide adenine dinucleotide NAD(H) dehydrogenase subunit A9 (NDUFA9) to augment complex I activity and maintaining basal energy production.^[^ ^38^ ^]^ Moreover, SIRT3 positively regulated succinate dehydrogenase activity by balancing mitochondrial NAD^+^ and NAD(H) levels.^[^ ^39^ ^]^ More remarkably, we found that SIRT3‐induced chondroprotective effect is associated with activation of intracellular antioxidant enzymes, of which selenoprotein GPX1 is significantly strengthened by SIRT3. Selenium deficiency is well correlated with the development of Kashin–Beck osteoarthropathy,^[^ ^40^ ^]^ an endemic disabling osteoarticular disease; whereas selenium supplementation benefits the prevention of Kashin–Beck in children.^[^ ^41^ ^]^ Selenophosphate synthetase 1, an endogenous selenium donor, is essential for the regulation of selenium metabolism, while its deficiency exacerbates OA development through oxidative stress injury.^[^ ^42^ ^]^ Subsequent studies will look into the roles of other sirtuins (SIRT5 or SIRT7) and selenoprotein (TXNRD1/2) in OA development.

Here we demonstrate that, for the first time, reservation of SIRT3 is of immediate importance in maintaining mitochondrial homeostasis and protecting against OA. However, a most recent work by Zhu et al. reported that cartilage‐specific deletion of SIRT3 ameliorates cartilage degeneration and synovial hyperplasia in high‐fat diet (HFD)‐induced metabolic OA model.^[^ ^43^ ^]^ The enhanced glycolysis and suppressed mitochondrial fatty acid metabolism induced by SIRT3 loss may serve as the leading causes. Differing from other cell types, owing to the avascular structure, in vivo, chondrocytes produce energy mainly though anaerobic respiration. Conventional views support that hypoxic environment benefit chondrocyte's natural functions.^[^ ^44^ ^]^ Although, unlike juvenile cartilage, chondrocytes in OA cartilage are unable to generate required energy, especially during the repair process. It is inferred that the transition from anaerobic to aerobic respiration may not always be deleterious, but perhaps as a compensatory mechanism to overcome energy insufficiency.^[^ ^45^ ^]^ On the other hand, HFD‐mediated OA development is dependent on metabolic alterations such as elevated fatty acid transport. Lipid and cholesterol accumulation are proposed as the contributing risk factors for OA development.^[^ ^46^ ^]^ Similar to our findings, SIRT3 exerts protective actions on articular cartilage in aging‐related OA model.^[^ ^12^ ^]^ Our gain‐of‐function experiments further suggested that activation of SIRT3 recouples the mitochondrial redox balance, a notable feature in OA,^[^ ^47^ ^]^ to attenuate chondrocytes injury. Therefore, the discrepancy of animal models and the corresponding molecular mechanisms may contribute to the Janus face of SIRT3 in OA. Interestingly, the regulatory effect of SIRT3 on bone homeostasis is also controversial, due to the SIRT3‐induced robust protection on osteoblast‐mediated bone formation and osteoclast‐induced bone resorption, simultaneously.^[^ ^48^, ^49^ ^]^ According to our µ‐CT reconstruction, bone mass and microarchitecture were augmented in subchondral bone plate in *Sirt3* ^−/−^ mice, implying another regulatory mechanism involving bone turnover. Meanwhile, over‐activated M1 macrophages and IL‐1*β* secretion in *Sirt3^−/−^* mice shed new light on mitigating OA through manipulating synovial inflammation and polarization. SUMO‐specific protease SENP1 activates SIRT3 via de‐SUMOylation to augment M2 macrophage polarization, through the accumulation of *α*KG in a glutaminolysis‐dependent manner.^[^ ^50^ ^]^ More specific information and the underlying mechanisms of SIRT3 in cartilage–bone–synovium crosstalk will be explored in our future work.

The upstream molecular mechanisms underlying SIRT3‐regulated cartilage mitochondrial steadiness and ECM equilibrium are still unknown. Conventional wisdom holds that adenosine 5′ monophosphate‐activated protein kinase (AMPK), an enzyme guardian of cellular metabolism, acts as the upstream sensor of SIRT3 in modulating energy balance and mitochondrial homeostasis. In mice, AMPK (*Ampkα1* ^−/−^) knockout reduces SIRT3 activity, deletes mtDNA integrity, and impairs mitochondrial function.^[^ ^51^ ^]^ Similarly, berberine‐induced AMPK*α* phosphorylation ameliorates joint structural damage and pain in post‐traumatic OA by simultaneously activating SIRT1 and SIRT3.^[^ ^52^ ^]^ The member of transcription coactivators, PPAR*γ* coactivator (PGC)‐1*α* is strongly associated with AMPK‐SIRT3 signaling pathway.^[^ ^53^ ^]^ Overexpression of PGC‐1*α* restores SIRT3 expression level in chronic kidney disease.^[^ ^54^ ^]^ Nucleus‐localized SIRT1 affects the expression of mitochondria‐enriched SIRT3 after oxygen and glucose deprivation through modulation of the PGC1‐dependent pathway. This implies that PGC‐1*α* plays a transporter or messenger role to transmit information between different organelles.^[^ ^55^ ^]^ Additionally, with improved methods to evaluate the transcriptome, non‐coding RNAs are coming to light in regulating proteins’ expression and activities.^[^ ^56^ ^]^ The miR‐195‐5p downregulates SIRT3 expression through direct 3′‐untranslated region (UTR) targeting in failing myocardium to enhance the acetylation of pyruvate dehydrogenase complex or ATP synthase (complex V).^[^ ^57^ ^]^ Further studies are needed to investigate the potential upstream signaling pathways on SIRT3. To achieve the goals of precision medicine in treating OA, several key components of biomedical research must be seamlessly integrated across several potential therapeutic targets. In the present study, a natural ingredient honokiol, which is derived from traditional Chinese medicine *HOUPU*, was used to motivate SIRT3.^[^ ^58^ ^]^ Our findings corroborated an upregulated SIRT3 expression in chondrocytes, as well as the reinforcement of mitochondrial function or preservation of cartilage matrix after honokiol treatment. Similarly, honokiol‐induced SIRT3 activation inhibits cardiac hypertrophic responses and attenuates pre‐existing cardiac hypertrophy in mice via augmentation of mitochondrial respiration and reduction of mitochondrial ROS production.^[^ ^59^ ^]^ A 3D printing of polyethylene glycol diacrylate/ECM hydrogel incorporating honokiol facilitates cartilage regeneration in an osteochondral defect model, supporting the innovative applied perspectives of honokiol in cartilage tissue engineering.^[^ ^60^ ^]^ Besides honokiol, multiple agonists of SIRT3 have been developed to combat mitochondrial function loss in various diseases. Flavonoid dihydromyricetin (DHM) supplementation mitigates nonalcoholic fatty liver disease by improving mitochondrial respiratory capacity and oxidative balance in hepatocytes via a SIRT3‐dependent mechanism.^[^ ^61^ ^]^ Meanwhile, DHM‐SIRT3 activation prevents chondrocytes from inflammatory cytokines‐induced ECM degradation and maintains mitochondrial metabolic equilibrium.^[^ ^62^ ^]^ Melatonin, *N*‐acetyl‐5‐methoxytryptamine, is a highly conserved indoleamine secreted from the pineal gland. Our previous studies systematically reported the pivotal role of the melatonin‐SIRT1 axis in controlling the fate of chondrocytes and antagonizing OA.^[^ ^63^ ^]^ Melatonin has a hepatoprotective effect on mitochondrial‐derived free radicals‐stimulated autophagic cell death, which is dependent on SIRT3/SOD2 pathway activation.^[^ ^64^ ^]^ Furthermore, the Warburg effect during lung cancer development could be reversed by melatonin‐SIRT3‐stimulated mitochondrial membrane polarization and the activities of complex I and IV in ETC.^[^ ^65^ ^]^ Given the benefits of recombinant proteins such as lack of off‐target side effects, recombinant SIRT3 protein‐based strategies are being designed and prepared in our experiments to further boost the mitochondrial activity of cartilage and open up new therapeutic options for OA treatment.

In conclusion, our research found a link between SIRT3‐induced mitochondrial deacetylation and the development of OA. The SIRT3 deletion and mitochondrial respiration dysfunction downstream of COX4I2 accelerated cartilage ECM destruction and OA progression. The natural agonist honokiol activated SIRT3 and reversed cartilage degeneration while maintaining ECM metabolic balance. Our findings highlight the functional importance of SIRT3 deacetylation machinery in mitochondrial homeostasis, providing insight into the novel therapeutic strategy for treating OA.

## 4 Experimental Section

### Ethics Statement

Articular cartilage samples were collected from consenting total knee arthroplasty patients. The ethical committee of the First Affiliated Hospital of Soochow University permitted this study (2022AE366). Moderate cartilage explants were harvested from six patients (three males and three females) while severe cartilage explants were harvested from six patients (two males and four females). The detailed information for all patients is presented in Table S1, Supporting Information. Experimental procedures involving animals were performed in accordance with guidelines of the Ethics Committee of Soochow University (SUDA20210406A02).

### Genetic Knockout Model

Global *SIRT3^−/−^* mice were purchased from GemPharmatech Co., Ltd. (Nanjing, China). Age‐matched WT littermates were used as control (CTRL) mice and all mice were of the C57BL/6J background. Genotyping of mice was performed using tail DNA according to the instructions of the Jackson Laboratory. All animals were provided with a standard diet and housed in pathogen‐free cages at a constant temperature and humidity.

### Establishment of Post‐Traumatic OA Mice Models

8‐week‐old *SIRT3* knockout mice (*n* = 6) and the corresponding littermate CTRL mice (*n* = 6) were subjected to DMM surgery to establish post‐traumatic OA models. Briefly, mice joint capsules were immediately incised after anesthesia, and the medial meniscotibial ligament transected to destabilize the meniscus without damaging other tissues. For the sham operation, incisions were performed without medial meniscus transection (*n* = 6). 8 weeks after surgery, all mice were sacrificed for collection of knee‐joint samples by cervical dislocation.

### Intra‐Articular Injection of Honokiol

To assess the potential therapeutic effects of SIRT3 against OA, a natural SIRT3 agonist (honokiol, HKL, Topscience, Shanghai, China) was intra‐articularly injected into joint cavities of sham or DMM‐induced OA mice twice a week. Mice injected with an equal volume of saline were used as controls. At 8 weeks after surgery, mice were sacrificed and their knee joints obtained for subsequent experiments.

### µ‐CT Imaging Analyses

Knee joint samples were scanned using micro‐CT (Skyscan 1176, Skyscan, Aartselaar, Belgium) with a high resolution of 9 µm at 50 kV/200 µA. The NRecon v1.6 and CTAn v1.13.8.1 softwares were used for data reconstruction. Regions of interest were defined as the 30 consecutive layers of medial tibial subchondral bone. Subchondral bone‐specific parameters included bone volume versus tissue volume ratio (%), trabecular thickness (Tb.Th, mm), and trabecular separation (Tb.Sp., mm^−1^). Scanned images from each group were subjected to the same thresholds to allow for 3D structural remodeling.

### Histological Analyses

Samples were fixed in 4% formalin (Jiancheng, Nanjing, China) for 48 h, decalcified in 0.5 m ethylenediaminetetraacetic acid (Yuanye, Shanghai, China) and embedded. Sections were deparaffinized in xylene, hydrated using graded ethanol, and stained with hematoxylin and eosin (H&E), safranin O‐fast green (S.O.), or toluidine blue (T.B.). The International Cartilage Repair Society cartilage lesion classification system, OARSI scoring system, and the ratio of HC versus CC were used to assess cartilage degeneration. The Krenn's synovitis scoring system was used to assess synovial activations. Each section was assessed by two blinded, independent graders (ZY and LY) and the mean score was used for statistical analysis.

### Immunohistochemistry and Immunofluorescence Analyses

After deparaffinization and rehydration, sections were soaked in citrate buffer (10 mm citric acid, pH = 6.0, Jiancheng) at 60°C for 12 h to repair the antigen for immunohistochemistry (IHC) and immunofluorescence (IF) staining. Sections were incubated with 3% hydrogen peroxide for 15 min and blocked with 1% sheep serum for 1 h. Then, they were incubated with specific primary antibodies: anti‐COLII (ab188570, Abcam, Cambridge, MA, USA), anti‐ACAN (A11691, Abclonal, Wuhan, China), anti‐MMP13 (A16920), anti‐ADAMTS5 (A2836), anti‐SIRT3 (A20805), and anti‐COX4I2 (ab70112) at 4 °C overnight. For IHC analysis, sections were incubated with horseradish peroxidase (HRP)‐conjugated secondary antibodies, stained with 3,3′‐diaminobenzidine (Vector Laboratories, Burlingame, CA, USA) and counterstained with hematoxylin. For IF analysis, sections were stained with Alexa 488 or Alexa 594 dye‐labeled secondary antibodies (Abcam), and with 4,6‐diamidino‐2‐phenylindole for nuclear counterstaining. Images were captured with a Zeiss Axiovert 40CFL microscope (Zeiss, Oberkochen, Germany). Positively stained cells were quantified using an ImageJ software (National Institutes of Health, Bethesda, MD, USA). The corresponding colocalization ratio based on the Pearson's correlation (Rr) and overlap coefficient (*R*) between SIRT3 and COX4I2 were calculated by ImageJ analysis.

### Cell Cultures

Articular chondrocytes were isolated from femoral heads, femoral condyles, and tibial plateaus of C57BL/6J mice. Mice cartilage were digested using 0.2% type II collagenase (Sigma‐Aldrich, St. Louis, MO, USA) at 37 °C overnight. Primary chondrocytes were cultured in Dulbecco's modified Eagle's medium:nutrient mixture F‐12 (Thermo Fisher Scientific, Waltham, MA, USA) supplemented with 10% fetal bovine serum and 1% penicillin–streptomycin (Thermo Fisher Scientific) at 37 °C in a 5% CO_2_ atmosphere. Subsequent experiments were performed on chondrocytes from passage one.

### Cell Treatments

To mimic the multidimensional arthritic environment in vitro, cells were treated with 10 ng mL^−1^ IL‐1*β* (Thermo Fisher Scientific), 10 ng mL^−1^ TNF‐*α* (Thermo Fisher Scientific), or 100 µm TBHP (Thermo Fisher Scientific) for 1 week, respectively. To establish the protective effects of SIRT3 against OA, cells were treated with 10 µm HKL with or without IL‐1*β* treatment. To determine the crosstalk between SIRT3 and COX4I2, cells were transfected with 100 nm *Sirt3*‐or *Cox4i2‐*targeting pcDNA3.1 vector using the Lipofectamine 3000 reagent (Thermo Fisher Scientific), as instructed by the manufacturers.

### Quantitative Reverse Transcription PCR

Total RNA were extracted using the TRIzol reagent (Thermo Fisher Scientific), and reverse transcribed into complementary DNA (cDNA) using the RevertAid H Minus First Strand cDNA synthesis kit (Takara, Tokyo, Japan). Quantitative reverse transcription PCR was performed using the iTap Universal SYBR Green Supermix kit (Bio‐Rad, Hercules, CA, USA). The amplification conditions were: initial denaturation at 95 °C for 10 min, followed by 40 cycles at 95 °C for 10 s, 60 °C for 30 s, and extension at 72 °C for 30 s. Relative expressions of target genes were determined using the comparative Ct (2^−ΔΔ^ *^Ct^*) method with *Gapdh* as the internal standard. The primer sequences used in this assay are shown in Table S2, Supporting Information.

### Western Blot Assay

Total cellular protein were extracted using with ice‐cold RIPA lysis buffer containing protease inhibitor cocktails (Sigma‐Aldrich) and their concentrations determined using the BCA Protein Assay Kit (Beyotime, Shanghai, China). Protein extracts were subjected to SDS‐PAGE and transferred to polyvinylidene fluoride membranes (Bio‐Red, Richmond, CA, USA). Membranes were incubated at 4 °C overnight in the presence of primary antibodies: anti‐COLII (ab188570), anti‐ACAN (A11691), anti‐MMP13 (A16920), anti‐ADAMTS5 (A2836), anti‐SIRT3 (A20805), anti‐COX4I2 (ab70112), or *α*‐Tubulin (AC007). Membranes were incubated with HRP‐conjugated secondary antibodies at room temperature and visualized using an enhanced chemiluminescence kit (Thermo Fisher Scientific). Gray values of the bands were quantified using the ImageJ software and normalized to corresponding expressions of *α*‐Tubulin.

### Measurement of Oxygen Consumption Rate

Oxygen consumption rate (OCR) was measured using the XF96 SeaHorse Analyzer (Seahorse Biosciences, North Billerica, MA, USA). The OCR rate was measured before and after the addition of inhibitors (2 µm oligomycin, a complex V inhibitor; 1 µm FCCP, a protonophore; and 1 µm antimycin A and rotenone, complex III and I inhibitors, respectively) to derive parameters of mitochondrial respiration. The calculation of basal respiration, ATP production, respiratory capacity, and respiratory reserve was performed using a Wave software (Agilent).

### Mitochondrial Membrane Potential (*ΔΨm*)

Mitochondrial membrane potential (*ΔΨm*) level was determined using JC‐1 staining kit (Beyotime). Chondrocytes was washed and incubated with 5 µm JC‐1 at 37 °C for 30 min. Fluorescence images were captured with a Zeiss Axiovert 40CFL microscope. The *ΔΨm* level was calculated by the red versus green fluorescence intensity ratio using ImageJ analysis.

### Reactive Oxygen Species

For intracellular ROS, chondrocytes were incubated with 10 µm DCFH‐DA (Beyotime). For mitochondrial ROS, cells were incubated with 5 µm MitoSOX Red mitochondrial superoxide indicator (Thermo Fisher Scientific). The fluorescent cell images were visualized using a Zeiss Axiovert 40CFL microscope. Fluorescence quantification was carried out using ImageJ.

### Co‐Immunoprecipitation Assay

Total proteins were extracted from cells transfected with or without *Sirt3*‐targeting pcDNA3.1 vector using Lipofectamine 3000 (Thermo Fisher Scientific). Cell lysates were precleared for 2 h at room temperature using protein A/G plus agarose and incubated at 4 °C overnight in the presence of anti‐SIRT3, anti‐COX4I2, or non‐specific IgG antibodies. Cells were supplemented with protein A/G plus agarose and incubated for 2 h. After washing three times, samples were boiled with SDS‐PDGE loading buffer and subjected to western blot.

### COX4I2 Acetylation Analysis

Total proteins were extracted from cells transfected with or without *Sirt3*‐targeting pcDNA3.1 vector using Lipofectamine 3000. After preclearing with protein A/G plus agarose (Thermo Fisher Scientific), cell lysates were immunoprecipitated at 4 °C overnight in the presence of the anti‐COX4I2 antibody. Cell lysates were supplemented with protein A/G plus agarose and incubated for 2 h. After washing three times, samples were boiled with SDS‐PAGE loading buffer and subjected to western blot.

### Label‐Free Proteomics

Proteins extracted from WT or *Sirt3* ^−/−^ mice articular chondrocytes were digested using trypsin. The digested peptides were desalted, dried, and resuspended in 0.1% formic acid. Then, peptides were analyzed using a nano‐scale liquid chromatographic tandem mass spectrometry (nLC‐MS) system (Easy‐nLC 1200, Thermo Fisher Scientific) coupled to a Q‐Exactive (Thermo Fisher Scientific). The scanning mode for mass spectrometry was data‐dependent acquisition mode and precursors from 350 to 1600 *m*/*z* were scanned for MS/MS.

### RNA Sequencing

The RNA sequencing of mice chondrocytes was performed by Affymetrix Gene Chip microarrays (Affymetrix, Santa Clara, CA, USA) after they had been treated with 10 ng mL^−1^ IL‐1*β* or PBS. The Agilent Bioanalyzer 2100 (Agilent Technologies, Santa Clara, CA, USA) was used to determine RNA expressions, which were then analyzed using the Significant Analysis of the Microarray software. Gene expressions with fold change >2 was considered valid. RNA sequencing was performed at Wekemo Tech Group Co., Ltd. (Shenzhen China).

### Molecular Docking

The molecular structure of honokiol was retrieved from PubChem Compound. The 3D coordinate of SIRT3 was downloaded from the PDB. All protein and molecular files were converted into PDBQT format with all water molecules excluded and polar hydrogen atoms were added. Molecular docking calculations were carried out using the Autodock Vina 1.2.2.

### Statistical Analysis

Data were presented as mean ± standard deviation (SD). Comparisons of means between and among groups were, respectively, performed using the two‐tailed Student's *t*‐test and two‐way analysis of variance followed by the Turkey's post‐test. Statistical significance was determined at *p < 0.05, **p < 0.01, or ***p < 0.001. Graph Pad Prism software 9.3 (GraphPad Software, Inc., La Jolla, CA, USA) was used for analyses.

### Role of Funding Source

The funders had no role in study design, data collection, interpretation and analysis, decision to publish, or preparation of the manuscript.

## Conflict of Interest

The authors declare no conflict of interest.

## Author Contributions

Y.Z., Y.L., and M.H. contributed equally to this work. All authors were involved in drafting the article or revising it critically for important intellectual content, and all authors approved the final version to be published. X.Z. had full access to all the data in the study and takes responsibility for the integrity of the data and the accuracy of the data analysis. Conception and design: Y.Z., F.H., and X.Z. Data acquisition: M.H., Y.L., X.X., J.L., and Y.S. Data analysis and interpretation: Y.X., Q.S., Z.Z., L.W., H.Y., and X.Z.

## Supporting information

Supporting Information

## Data Availability

The data that support the findings of this study are available from the corresponding author upon reasonable request.

## References

[1] D. J. Hunter , L. March , M. Chew , Lancet 2020, 396, 1711. 33159851 10.1016/S0140-6736(20)32230-3

[2] L. Sharma , N. Engl. J. Med. 2021, 384, 51. 33406330 10.1056/NEJMcp1903768

[3] G. Wang , S. Chen , Z. Xie , S. Shen , W. Xu , W. Chen , Ann. Rheum. Dis. 2020, 79, 1111. 32409323 10.1136/annrheumdis-2019-216911 PMC7392491

[4] F. Kraus , K. Roy , T. J. Pucadyil , M. T. Ryan , Nature 2021, 590, 57. 33536648 10.1038/s41586-021-03214-x

[5] L. Gambarotto , S. Metti , M. Chrisam , C. Cerqua , P. Sabatelli , A. Armani , J. Cachexia Sarcopenia Muscle 2022, 13, 2211. 35593053 10.1002/jcsm.13010 PMC9434724

[6] X. Wei , Z. Zheng , Z. Feng , L. Zheng , S. Tao , B. Zheng , EMBO Mol. Med. 2022, 14, 15373. 10.15252/emmm.202115373 PMC9260208 35611810

[7] F. J. Blanco , A. M. Valdes , I. Rego‐Pérez , Nat. Rev. Rheumatol. 2018, 14, 327. 29670212 10.1038/s41584-018-0001-0

[8] Y. Zhang , M. Hou , Y. Liu , T. Liu , X. Chen , Q. Shi , J. Pineal Res. 2022, 73, e12815. 35726138 10.1111/jpi.12815

[9] L. Zhang , X. Chen , P. Cai , H. Sun , S. Shen , B. Guo , Adv. Mater. 2022, 34, 2202715. 10.1002/adma.202202715 35671349

[10] M. D. Hirschey , T. Shimazu , E. Goetzman , E. Jing , B. Schwer , D. B. Lombard , Nature 2010, 464, 121. 20203611 10.1038/nature08778 PMC2841477

[11] R. Tao , M. C. Coleman , J. D. Pennington , O. Ozden , S. H. Park , H. Jiang , Mol. Cell 2010, 40, 893. 21172655 10.1016/j.molcel.2010.12.013 PMC3266626

[12] Y. Fu , M. Kinter , J. Hudson , K. M. Humphries , R. S. Lane , J. R. White , Arthritis Rheumatol. 2016, 68, 1887. 26866626 10.1002/art.39618 PMC5331855

[13] S. Someya , W. Yu , W. C. Hallows , J. Xu , J. M. Vann , C. Leeuwenburgh , Cell 2010, 143, 802. 21094524 10.1016/j.cell.2010.10.002 PMC3018849

[14] J. F. Hevler , R. Z. Chiozzi , A. Cabrera‐Orefice , U. Brandt , S. Arnold , A. J. R. Heck , Proc. Natl. Acad. Sci. U. S. A. 2021, 118, e2106950118. 34548399 10.1073/pnas.2106950118 PMC8488679

[15] N. Sommer , M. Hüttemann , O. Pak , S. Scheibe , F. Knoepp , C. Sinkler , Circ. Res. 2017, 121, 424. 28620066 10.1161/CIRCRESAHA.116.310482 PMC5544581

[16] F. Berenbaum , T. M. Griffin , R. Liu‐Bryan , Arthritis Rheumatol. 2017, 69, 9. 27564539 10.1002/art.39842 PMC5341385

[17] F. J. Blanco , I. Rego , C. Ruiz‐Romero , Nat. Rev. Rheumatol. 2011, 7, 161. 21200395 10.1038/nrrheum.2010.213

[18] S. Zhou , Z. Cui , J. P. Urban , Arthritis Rheumatol. 2004, 50, 3915. 10.1002/art.20675 15593204

[19] Y. Wang , X. Zhao , M. Lotz , R. Terkeltaub , R. Liu‐Bryan , Arthritis Rheumatol. 2015, 67, 2141. 25940958 10.1002/art.39182 PMC4519411

[20] P. L. de Figueroa , M. K. Lotz , F. J. Blanco , B. Caramés , Arthritis Rheumatol. 2015, 67, 966. 25605458 10.1002/art.39025 PMC4380780

[21] O. R. Baris , S. Ederer , J. F. Neuhaus , J. C. von Kleist‐Retzow , C. M. Wunderlich , M. Pal , Cell Metab. 2015, 21, 667. 25955204 10.1016/j.cmet.2015.04.005

[22] T. Holzer , K. Probst , J. Etich , M. Auler , V. S. Georgieva , B. Bluhm , J. Cell Biol. 2019, 218, 1853. 31085560 10.1083/jcb.201809056 PMC6548139

[23] E. Maneiro , M. J. López‐Armada , M. C. de Andres , B. Caramés , M. A. Martín , A. Bonilla , P. D. Hoyo , F. Galdo , J. Arenas , F. J. Blanco , Ann. Rheum. Dis. 2005, 64, 388. 15708893 10.1136/ard.2004.022152 PMC1755391

[24] R. Fukuda , H. Zhang , J. W. Kim , L. Shimoda , C. V. Dang , G. L. Semenza , Cell 2007, 129, 111. 17418790 10.1016/j.cell.2007.01.047

[25] K. Čunátová , D. P. Reguera , M. Vrbacký , E. Fernández‐Vizarra , S. Ding , I. M. Fearnley , Cells 2021, 10, 369. 33578848 10.3390/cells10020369 PMC7916595

[26] E. Maneiro , M. A. Martín , M. C. de Andres , M. J. López‐Armada , J. L. Fernández‐Sueiro , P. del Hoyo , F. Galdo , J. Arenas , F. J. Blanco , Arthritis Rheum. 2003, 48, 700. 12632423 10.1002/art.10837

[27] Z. Jin , W. Wei , M. Yang , Y. Du , Y. Wan , Cell Metab. 2014, 20, 483. 25130399 10.1016/j.cmet.2014.07.011 PMC4156549

[28] F. P. Altman , Ann. Rheum. Dis. 1981, 40, 303. 7247474 10.1136/ard.40.3.303 PMC1000767

[29] X. Zhu , F. Chen , K. Lu , A. Wei , Q. Jiang , W. Cao , Ann. Rheum. Dis. 2019, 78, 1420. 31239244 10.1136/annrheumdis-2018-214940

[30] R. H. Houtkooper , E. Pirinen , J. Auwerx , Nat. Rev. Mol. Cell Biol. 2012, 13, 225. 22395773 10.1038/nrm3293 PMC4872805

[31] I. H. Lee , L. Cao , R. Mostoslavsky , D. B. Lombard , J. Liu , N. E. Bruns , Proc. Natl. Acad. Sci. U. S. A. 2008, 105, 3374. 18296641 10.1073/pnas.0712145105 PMC2265142

[32] T. Matsuzaki , T. Matsushita , K. Takayama , T. Matsumoto , K. Nishida , R. Kuroda , Ann. Rheum. Dis. 2014, 73, 1397. 23723318 10.1136/annrheumdis-2012-202620

[33] F. Niederer , C. Ospelt , F. Brentano , M. O. Hottiger , R. E. Gay , S. Gay , Ann. Rheum. Dis. 2011, 70, 1866. 21742641 10.1136/ard.2010.148957

[34] Y. Kanfi , S. Naiman , G. Amir , V. Peshti , G. Zinman , L. Nahum , Nature 2012, 483, 218. 22367546 10.1038/nature10815

[35] R. Mostoslavsky , K. F. Chua , D. B. Lombard , W. W. Pang , M. R. Fischer , L. Gellon , Cell 2006, 124, 315. 16439206 10.1016/j.cell.2005.11.044

[36] K. Nagai , T. Matsushita , T. Matsuzaki , K. Takayama , T. Matsumoto , R. Kuroda , Osteoarthritis Cartilage 2015, 23, 1412. 25819580 10.1016/j.joca.2015.03.024

[37] E. Jing , B. T. O'Neill , M. J. Rardin , A. Kleinridders , O. R. Ilkeyeva , S. Ussar , Diabetes 2013, 62, 3404. 23835326 10.2337/db12-1650 PMC3781465

[38] B. H. Ahn , H. S. Kim , S. Song , I. H. Lee , J. Liu , A. Vassilopoulos , Proc. Natl. Acad. Sci. U. S. A. 2008, 105, 14447. 18794531 10.1073/pnas.0803790105 PMC2567183

[39] I. Masgras , G. Cannino , F. Ciscato , C. Sanchez‐Martin , F. B. Darvishi , F. Scantamburlo , Cell Death Differ. 2022, 29, 1996. 35393510 10.1038/s41418-022-00991-4 PMC9525706

[40] R. Moreno‐Reyes , C. Suetens , F. Mathieu , F. Begaux , D. Zhu , M. T. Rivera , M. Boelaert , J. Nève , N. Perlmutter , J. Vanderpas , N. Engl. J. Med. 1998, 339, 1112. 9770558 10.1056/NEJM199810153391604

[41] K. Zou , G. Liu , T. Wu , L. Du , Osteoarthritis Cartilage 2009, 17, 144. 18693119 10.1016/j.joca.2008.06.011

[42] D. Kang , J. Lee , J. Jung , B. A. Carlson , M. J. Chang , C. B. Chang , S. B. Kang , B. C. Lee , V. N. Gladyshev , D. L. Hatfield , B. J. Lee , J. H. Kim , Nat. Commun. 2022, 13, 779. 35140209 10.1038/s41467-022-28385-7 PMC8828855

[43] S. Zhu , E. L. Donovan , D. Makosa , P. Mehta‐D'souza , A. Jopkiewicz , A. Batushansky , D. Cortassa , A. D. Simmons , E. B. P. Lopes , M. Kinter , T. M. Griffin , J. Bone Miner. Res. 2022, 37, 2531. 36214465 10.1002/jbmr.4721 PMC10091721

[44] B. D. Markway , G. K. Tan , G. Brooke , J. E. Hudson , J. J. Cooper‐White , M. R. Doran , Cell Transplant. 2010, 19, 29. 19878627 10.3727/096368909X478560

[45] M. Arra , G. Swarnkar , K. Ke , J. E. Otero , J. Ying , X. Duan , T. Maruyama , M. F. Rai , R. J. O'Keefe , G. Mbalaviele , J. Shen , Y. Abu‐Amer , Nat. Commun. 2020, 11, 3427. 32647171 10.1038/s41467-020-17242-0 PMC7347613

[46] C. Cao , Y. Shi , X. Zhang , Q. Li , J. Zhang , F. Zhao , Q. Meng , W. Dai , Z. Liu , W. Yan , X. Duan , J. Zhang , X. Fu , J. Cheng , X. Hu , Y. Ao , Nat. Commun. 2022, 13, 7139. 36414669 10.1038/s41467-022-34830-4 PMC9681739

[47] Z. Lv , J. Han , J. Li , H. Guo , Y. Fei , Z. Sun , J. Dong , M. Wang , C. Fan , W. Li , Y. Xie , W. Sun , J. Chen , Y. Liu , F. Chen , Z. Liu , A. Liu , R. Wu , X. Xu , W. Yan , Q. Jiang , S. Ikegawa , X. Chen , D. Shi , eBioMedicine 2022, 84, 104258. 36137413 10.1016/j.ebiom.2022.104258 PMC9494174

[48] J. Gao , Z. Feng , X. Wang , M. Zeng , J. Liu , S. Han , J. Xu , L. Chen , K. Cao , J. Long , Z. Li , W. Shen , J. Liu , Cell Death Differ. 2018, 25, 229. 28914882 10.1038/cdd.2017.144 PMC5762839

[49] W. Ling , K. Krager , K. K. Richardson , A. D. Warren , F. Ponte , N. Aykin‐Burns , S. C. Manolagas , M. Almeida , H. N. Kim , JCI Insight 2021, 6, e146728. 33878033 10.1172/jci.insight.146728 PMC8262324

[50] W. Zhou , G. Hu , J. He , T. Wang , Y. Zuo , Y. Cao , Q. Zheng , J. Tu , J. Ma , R. Cai , Y. Chen , Q. Fan , B. Dong , H. Tan , Q. Wang , W. Xue , J. Cheng , Cell Rep. 2022, 39, 110660. 35417703 10.1016/j.celrep.2022.110660

[51] L. Y. Chen , Y. Wang , R. Terkeltaub , R. Liu‐Bryan , Osteoarthritis Cartilage 2018, 26, 1539. 30031925 10.1016/j.joca.2018.07.004 PMC6202232

[52] J. Li , Y. Wang , D. Chen , R. Liu‐Bryan , Osteoarthritis Cartilage 2022, 30, 160. 34687898 10.1016/j.joca.2021.10.004 PMC8712393

[53] H. Amano , A. Chaudhury , C. Rodriguez‐Aguayo , L. Lu , V. Akhanov , A. Catic , Cell Metab. 2019, 29, 1274. 30930169 10.1016/j.cmet.2019.03.001 PMC6657508

[54] H. Feng , J. Y. Wang , B. Yu , X. Cong , W. G. Zhang , L. Li , Antioxid. Redox Signaling 2019, 31, 75. 10.1089/ars.2018.7620 30829051

[55] T. Chen , S. H. Dai , X. Li , P. Luo , J. Zhu , Y. H. Wang , Redox Biol. 2018, 14, 229. 28965081 10.1016/j.redox.2017.09.016 PMC5633840

[56] D. Andergassen , J. L. Rinn , Nat. Rev. Genet. 2022, 23, 229. 34837040 10.1038/s41576-021-00427-8

[57] X. Zhang , R. Ji , X. Liao , E. Castillero , P. J. Kennel , D. L. Brunjes , Circulation 2018, 137, 2052. 29330215 10.1161/CIRCULATIONAHA.117.030486 PMC6449058

[58] W. M. Qu , X. F. Yue , Y. Sun , K. Fan , C. R. Chen , Y. P. Hou , Br. J. Pharmacol. 2012, 167, 587. 22537192 10.1111/j.1476-5381.2012.02010.x PMC3449263

[59] V. B. Pillai , S. Samant , N. R. Sundaresan , H. Raghuraman , G. Kim , M. Y. Bonner , Nat. Commun. 2015, 6, 6656. 25871545 10.1038/ncomms7656 PMC4441304

[60] S. Zhu , P. Chen , Y. Chen , M. Li , C. Chen , H. Lu , Am. J. Sports Med. 2020, 48, 2808. 32762553 10.1177/0363546520941842

[61] X. Zeng , J. Yang , O. Hu , J. Huang , L. Ran , M. Chen , Antioxid. Redox Signaling 2019, 30, 163. 10.1089/ars.2017.7172 29310441

[62] J. Wang , K. Wang , C. Huang , D. Lin , Y. Zhou , Y. Wu , Int. J. Biol. Sci. 2018, 14, 1873. 30443190 10.7150/ijbs.27746 PMC6231225

[63] X. Zhou , Y. Zhang , M. Hou , H. Liu , H. Yang , X. Chen , J. Bone Miner. Res. 2022, 37, 1056. 35147250 10.1002/jbmr.4527

[64] H. Pi , S. Xu , R. J. Reiter , P. Guo , L. Zhang , Y. Li , Autophagy 2015, 11, 1037. 26120888 10.1080/15548627.2015.1052208 PMC4590599

[65] X. Chen , B. Hao , D. Li , R. J. Reiter , Y. Bai , B. Abay , J. Pineal Res. 2021, 71, e12755. 34214200 10.1111/jpi.12755

