# Supplementary material for: Reprogramming of Mitochondrial Respiratory Chain Complex by Targeting SIRT3‐COX4I2 Axis Attenuates Osteoarthritis Progression
Source: Adv Sci (Weinh). 2023 Jan 22;10(10):2206144. doi: 10.1002/advs.202206144 (PMC10074136; doi:10.1002/advs.202206144)
Supplement: Supplementary file 1 — Supporting Information [file ADVS-10-2206144-s001.pdf]

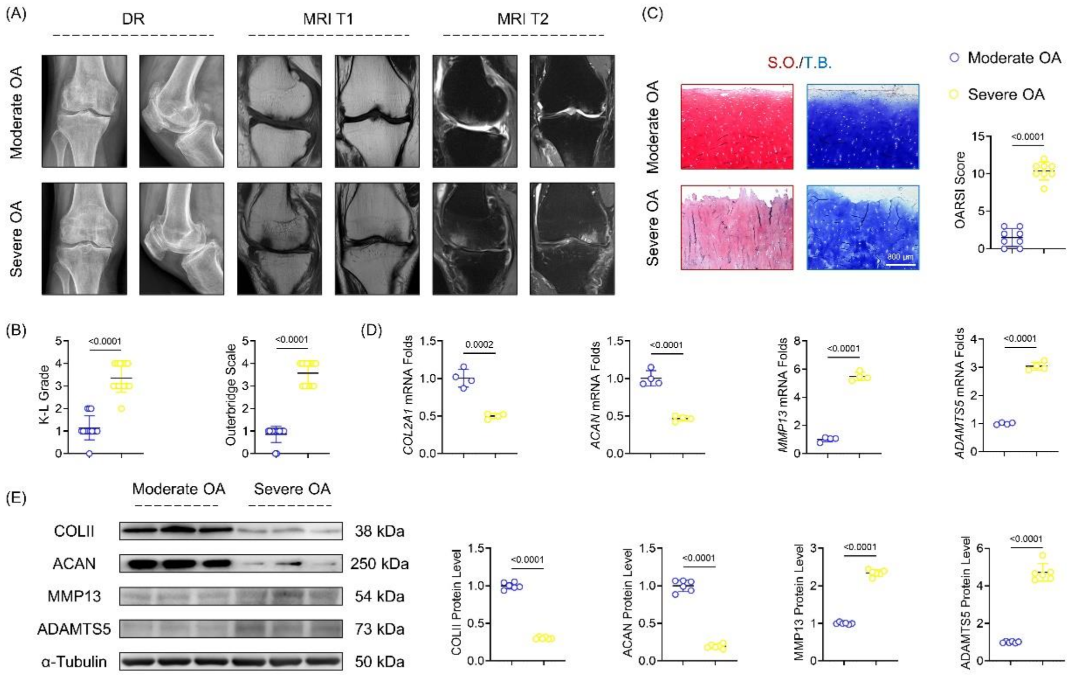

Supplementary Figure 1 The development of OA in human patients was accompanied with ECM metabolic disturbance. (A) Digital Radiography (DR) and Magnetic Resonance Imaging (MRI T1 or T2 weighted) of clinical moderate or severe OA patients. (B) Assessment of the severity OA according to Kellgren and Lawrence system or Outerbridge scale. (C) Representative images of Safranin O-Fast Green (S.O.) or Toluidine Blue (T.B.) staining of human cartilage samples and quantitative Analysis of cartilage erosion according to Osteoarthritis Research Society International (OARSI) scale. (D-E) Quantitative analysis of cartilage ECM mRNA and protein levels between moderate and severe OA samples. Data are presented as mean ± SD of at least three independent assays for each experiment. Statistically significant differences between groups are set at $p < 0.05$.

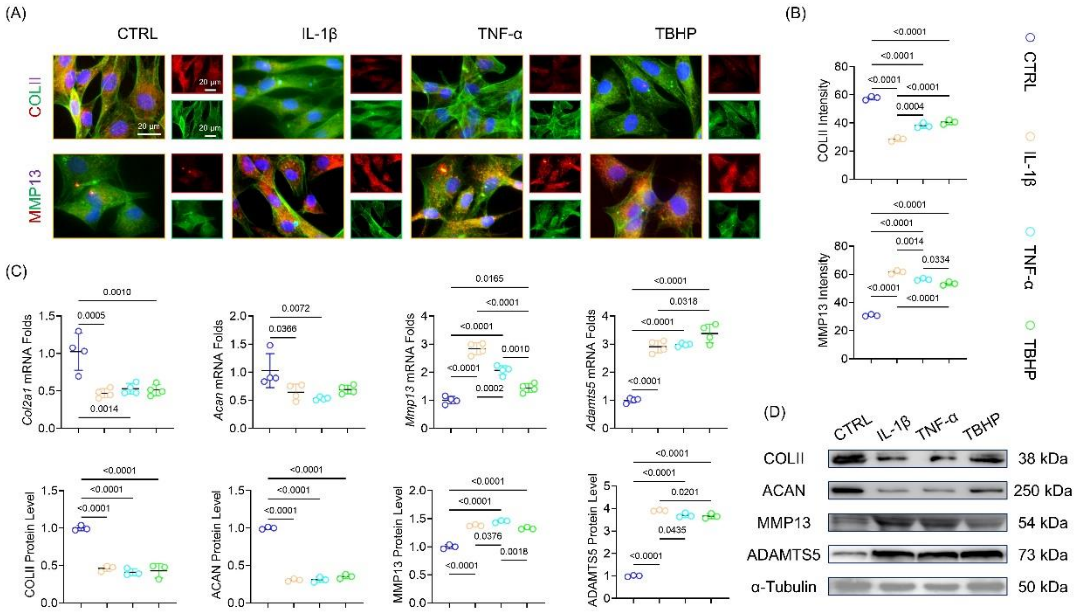

Supplementary Figure 2 An *in vitro* arthritic environment was established by treatment with three classical proinflammatory factors. (A-B) Immunofluorescence images and intensities of COLII or MMP13 in proinflammatory cytokine-treated mice chondrocytes. (C) Transcript levels of *Col2a1*, *Acan*, *Mmp13*, and *Adamts5* were determined via RT-PCR assays. (D) Protein levels of COLII, ACAN, MMP13, and ADAMTS5 were determined via Western blotting. Data are presented as mean ± SD of at least three independent assays for each experiment. Statistically significant differences between groups are set at $p < 0.05$.

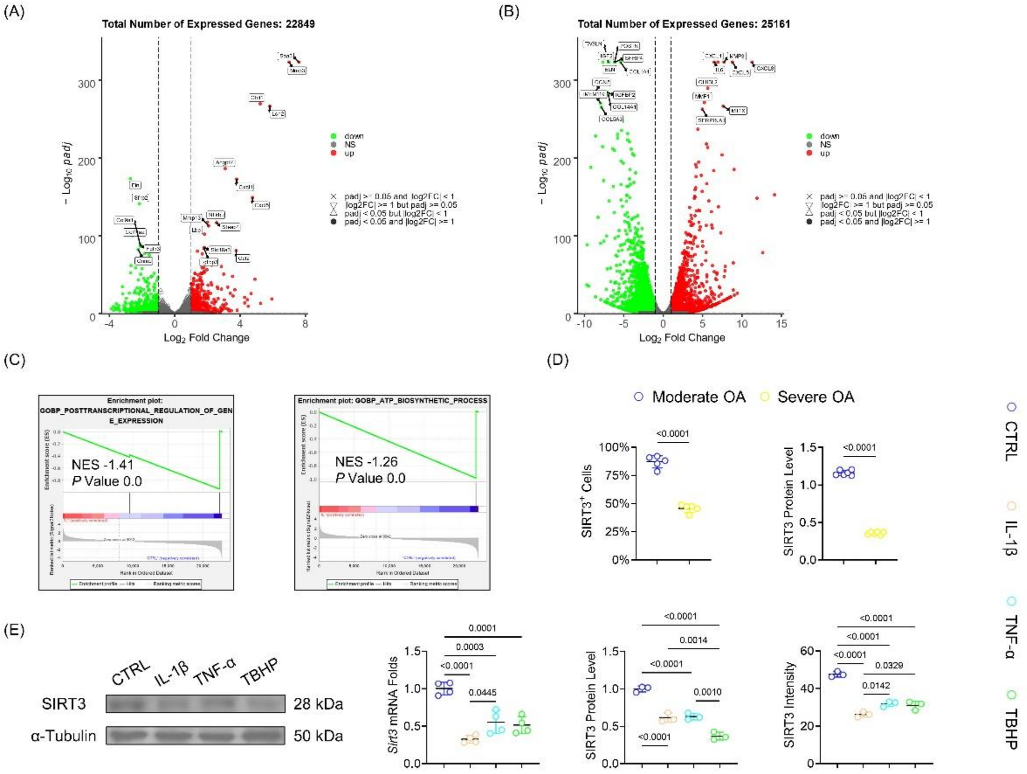

Supplementary Figure 3 Transcriptome analysis of human or mice OA cartilage samples. (A-B) The volcano map for differentially expressed genes. (C) Gene Set Enrichment Analysis (GSEA) of post-transcriptional regulation and ATP biogenesis. (D) The quantification of SIRT3-positive cells in human OA cartilage. (E-F) Gene and protein expressions of SIRT3 after treatment with pro-inflammatory cytokines. Data are presented as mean ± SD of at least three independent assays for each experiment. Statistically significant differences between groups are set at $p < 0.05$.

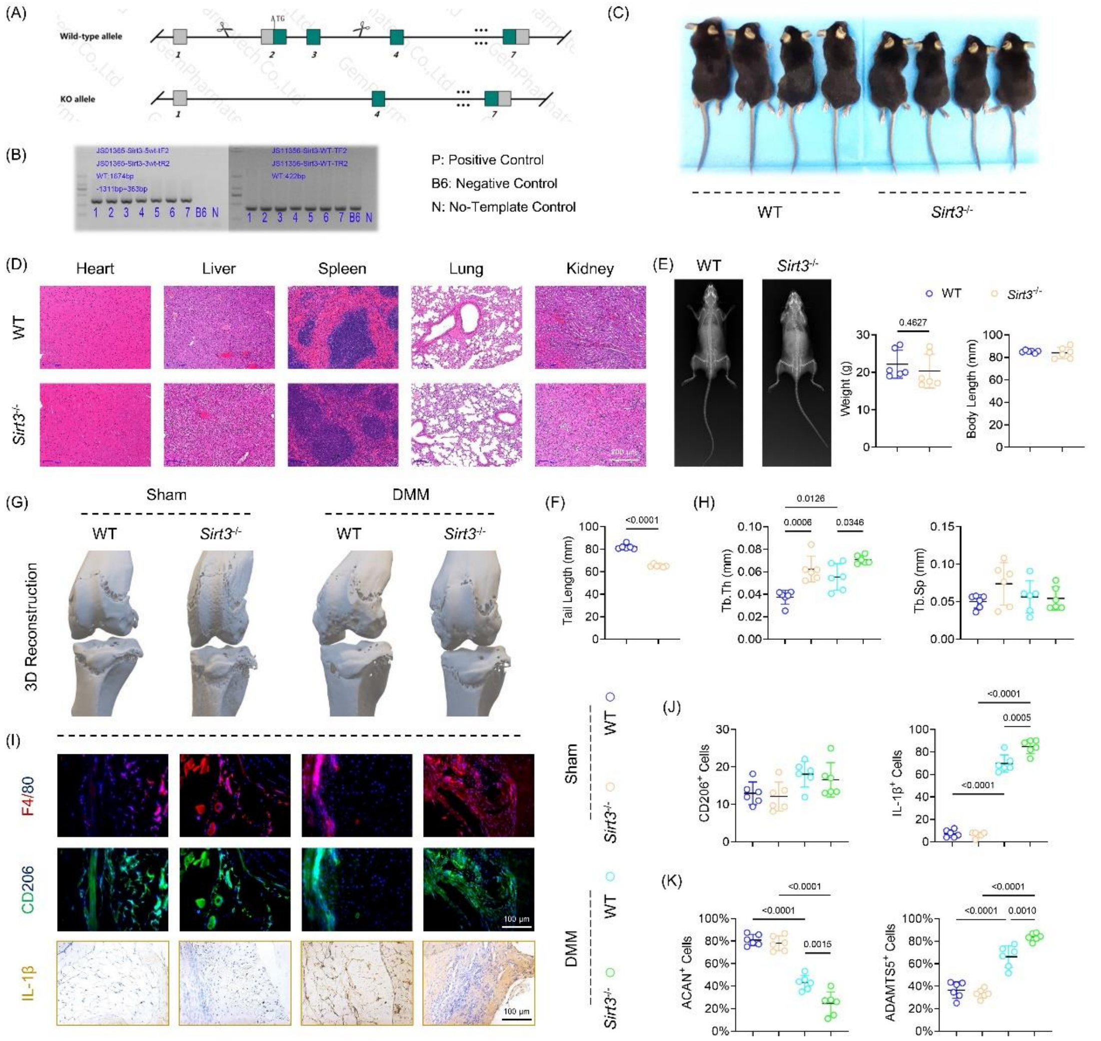

Supplementary Figure 4 SIRT3 knockout accelerated cartilage destruction and subchondral bone sclerosis. (A-B) Construction strategies and validation of SIRT3 global knockout in C57/BL6J mice. (C-D) Gross and bone morphologies of WT or *Sirt3*$^{-/-}$ mice (n=6). (E) Representative histological images in major organs (heart, liver, spleen, lung, and kidneys) and quantification of weight or body weight (n=6). (F) Quantification of tail length of WT or *Sirt3*$^{-/-}$ mice (n=6). (G-H) Surface reconstructions of knee joints and quantification of trabecular thickness (Tb.Th, mm) or trabecular separation (Tb.Sp., mm^{-1}) (n=6). (I-J) Representative images of immunofluorescence of F4/80 (top) and CD206 (middle), and IL-1β (bottom) in the synovium and quantification of CD206 or IL-1β-positive cells (n=6). (K)

Quantification of ACAN or ADAMTS5-positive chondrocytes (n=6). Data are presented as mean ± SD. Statistically significant differences between groups were determined at a threshold of $p < 0.05$.

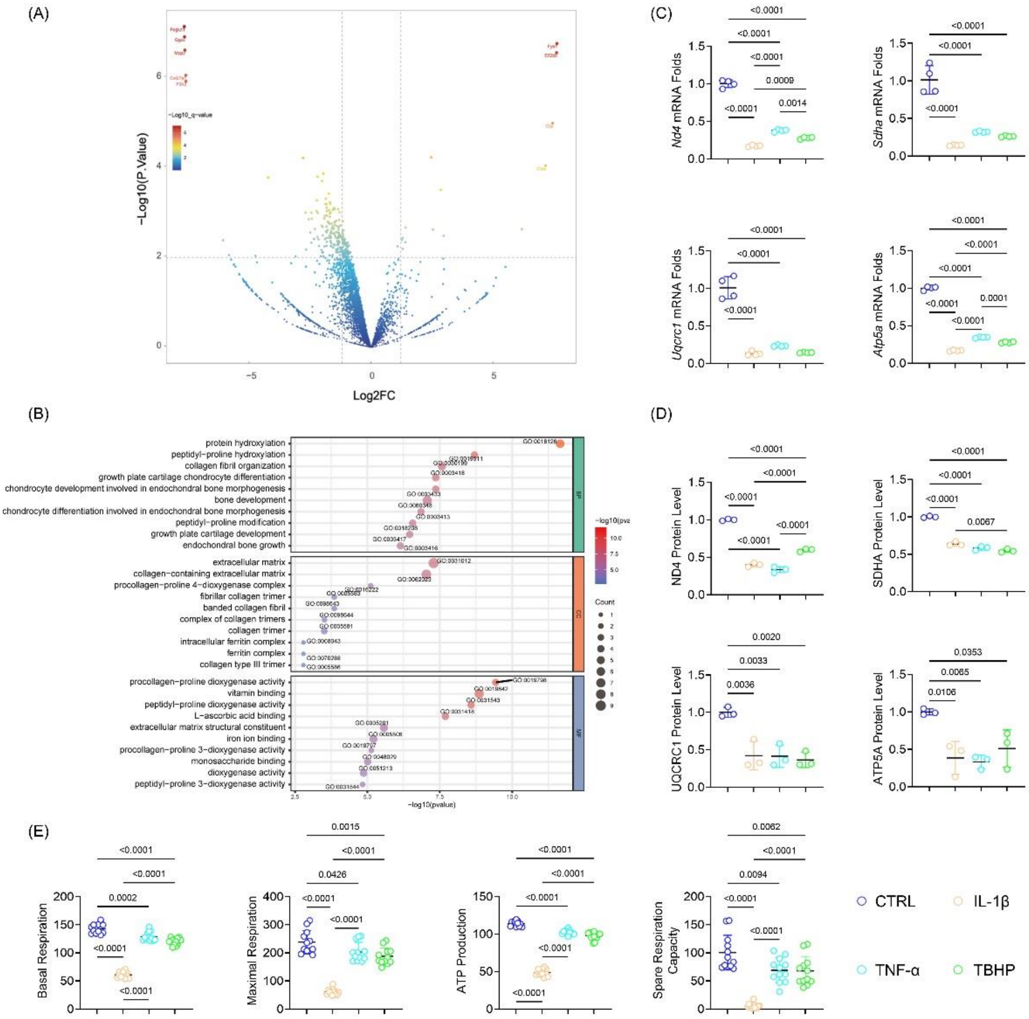

Supplementary Figure 5 SIRT3 knockout disrupted cartilage ECM and impaired MRC functions. (A) Volcano map for differentially expressed proteins between WT or *Sirt3*$^{-/-}$ mice. (B) GO enrichment analysis of differentially expressed proteins. (C) Transcript levels of *Nd4*, *Sdha*, *Uqcrc1*, *Cox4i2*, and *Atp5a* were determined via RT-PCR assays. (D) Quantitative protein levels of MT-ND4, SDHA, UQCRC1, COX4I2, and ATP5A. (E) Basal respiration, maximal respiration, ATP production, and spare respiration capacity expressed as pmol/min, normalized to protein concentration. Data are presented as mean ± SD of at least three independent assays for each experiment. Statistically significant differences between groups are set at $p < 0.05$.

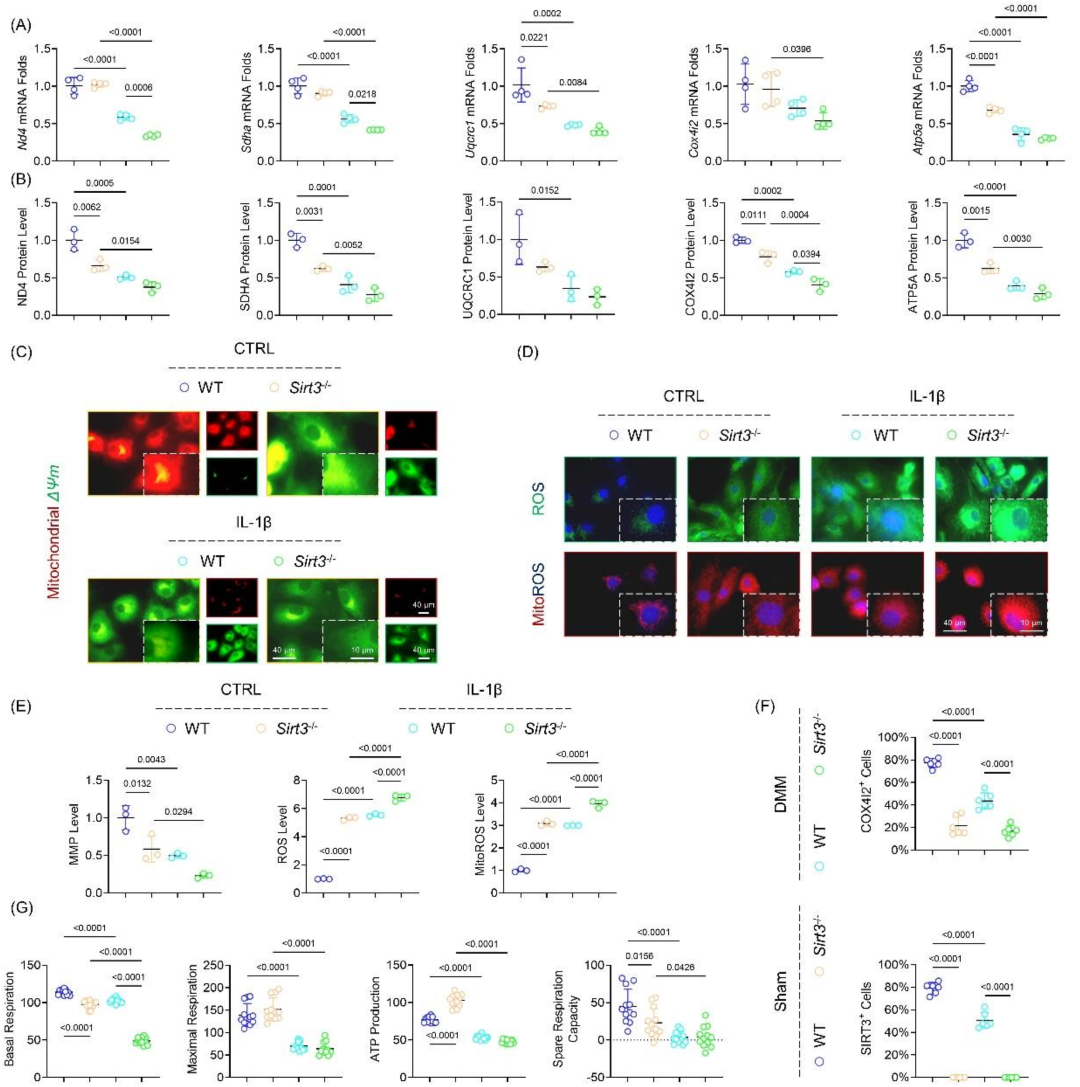

Supplementary Figure 6 Reservation of SIRT3 was essential for maintenance of MRC homeostasis. (A) Transcript levels of *Nd4*, *Sdha*, *Uqcrc1*, *Cox4i2* and *Atp5a* were determined via RT-PCR assays. (B) Quantitative protein levels of MT-ND4, SDHA, UQCRC1, COX4I2 and ATP5A. (C) The mitochondrial membrane potential was determined using a JC-1 assay. (D-E) Intracellular or mitochondrial reactive oxygen species (ROS) level was determined using DCFDA or mitoSOX dye. (F) Quantification of SIRT3 or COX4I2-positive cells in WT or *Sirt3*$^{-/-}$ mice. (G) Basal respiration, maximal respiration, ATP production, and spare

respiration capacity expressed as pmol/min, normalized to protein concentration. Data are presented as mean ± SD of at least three independent assays for each experiment. Statistically significant differences between groups are set at $p < 0.05$.

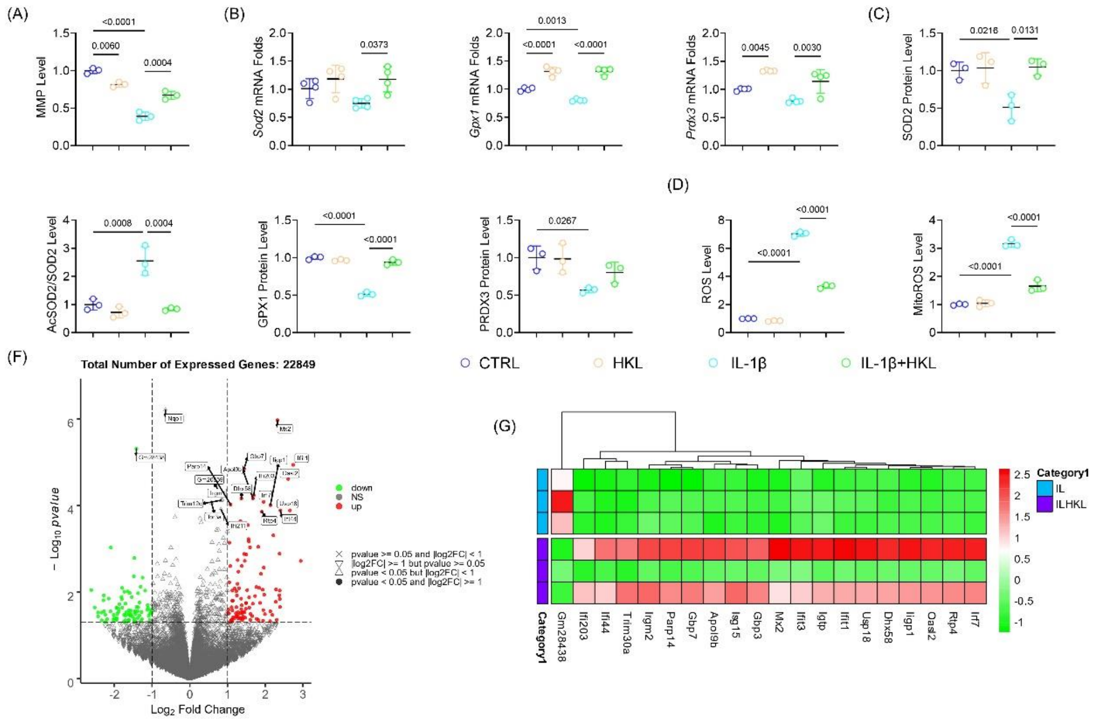

Supplementary Figure 7 Honokiol alleviated pro-inflammatory cytokine-induced oxidative stress. (A) Quantification of the mitochondrial membrane potential level. (B) Transcript levels of *Sod2*, *Gpx1*, and *Prdx3* were determined by RT-PCR assays. (C) Quantitative protein levels of SOD2, Ac-SOD2, GPX1, and PRDX3. (D) Quantification of the intracellular or mitochondrial ROS level. (F-G) Heatmap or volcano map for differentially expressed genes. Data are presented as mean ± SD of at least three independent assays for each experiment. Statistically significant differences between groups are set at $p < 0.05$.

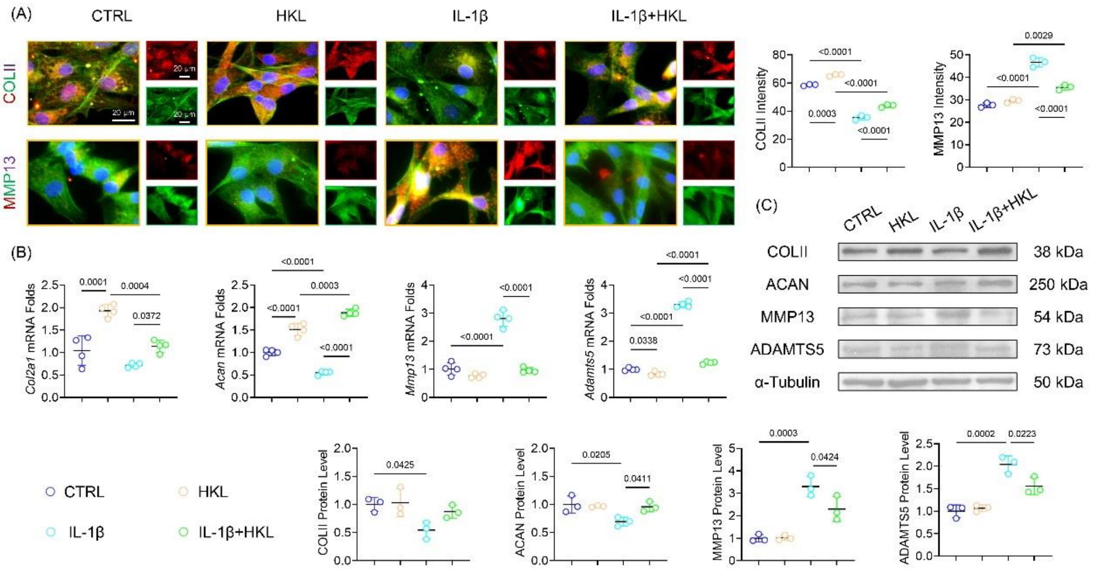

Supplementary Figure 8 Honokiol rescued pro-inflammatory cytokine-induced oxidative stress and ECM degradation. (A) The ECM anabolism COLII or catabolism MMP13 were determined by fluorescent immunostaining. (B-C) The mRNA or proteins levels of COLII, ACAN, MMP13, and ADAMTS5 were respectively determined by RT-PCR or western blot assays. Data are presented as mean ± SD of at least three independent assays for each experiment. Statistically significant differences between groups are set at $p < 0.05$.

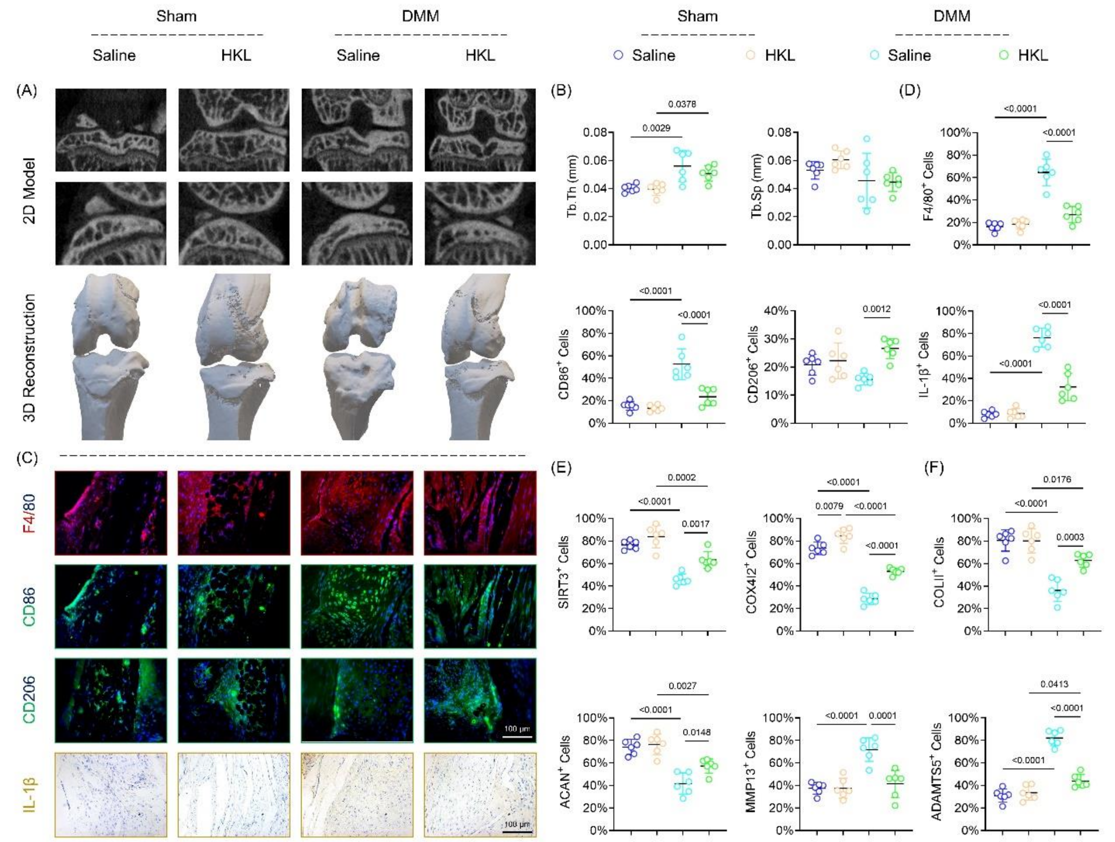

Supplementary Figure 9 Intra-articular injection of honokiol ameliorated cartilage degeneration. (A-B) Two-dimensional or surface reconstructions of knee joints and quantification of trabecular thickness (Tb.Th, mm) or trabecular separation (Tb.Sp., mm^{-1}) (n=6). (C-D) Representative images of immunofluorescence of F4/80 (top), CD86 and CD206 (middle), and IL-1β (bottom) in the synovium and quantification of F4/80, CD86, CD206, or IL-1β-positive cells (n=6). (E) Quantification of SIRT3 and COX4I2-positive chondrocytes. (F) Quantification of COLII, ACAN, MMP13, or ADAMTS5-positive chondrocytes (n=6). Data are presented as mean ± SD of six independent assays for each experiment. Statistically significant differences between groups were determined at a threshold of $p < 0.05$.

Supplementary Table 1. Basic information of clinical OA patients.

**Patients Number**	**Diagnosis**	**Gender**	**Age**
1	Moderate osteoarthritis	Female	56
2	Moderate osteoarthritis	Female	64
3	Severe osteoarthritis	Male	68
4	Moderate osteoarthritis	Male	72
5	Moderate osteoarthritis	Female	70
6	Severe osteoarthritis	Female	76
7	Severe osteoarthritis	Female	69
8	Severe osteoarthritis	Female	74
9	Moderate osteoarthritis	Male	68
10	Severe osteoarthritis	Male	77
11	Moderate osteoarthritis	Male	72
12	Severe osteoarthritis	Female	75

Supplementary Table 2. Primers used for real-time PCR

Gene	Forward Primer sequence (5'-3')	Reverse Primer sequence (5'-3')
Mmu-Col2a1	GGGAATGTCCTCTGCGATGAC	GAAGGGGATCTCGGGGTTG
Mmu-Acan	GTGGAGCCGTGTTTCCAAG	AGATGCTGTTGACTCGAACCT
Mmu-Mmp13	CTTCTTCTTGTTGAGCTGGACTC	CTGTGGAGGTCACTGTAGACT
Mmu-Adamts5	GGAGCGAGGCCATTTACAAC	CGTAGACAAGGTAGCCCACTTT
Mmu-Nd4	CTCCTCAGACCCCCTATCCA	AAATCCCTGCGTTTAGGCGT
Mmu-Sdha	GGAACACTCCAAAAACAGACCT	CCACCACTGGGTATTGAGTAGAA
Mmu-Uqcrc1	AGACCCAGGTCAGCATCTTG	GCCGATTCTTTGTTCCCTTGA
Mmu-Cox4i2	CTGCCCGGAGTCTGGTAATG	CGTAGCAGTCAACGTAGGGG
Mmu-Atp5a	TCTCCATGCCTCTAACACTCG	CCAGGTCAACAGACGTGTCAG
Mmu-Sod2	CAGACCTGCCTTACGACTATGG	CTCGGTGGCGTTGAGATTGTT
Mmu-Gpx1	CCACCGTGTATGCCTTCTCC	AGAGAGACGCGACATTCTCAAT
Mmu-Prdx3	GGTTGCTCGTCATGCAAGTG	CCACAGTATGTCTGTCAAACAGG
Mmu-Gapdh	AATGGATTTGGACGCATTGGT	TTTGCACTGGTACGTGTTGAT
Mmu-Sirt1	TGATTGGCACCGATCCTCG	CCACAGCGTCATATCATCCAG
Mmu-Sirt2	GCGGGTATCCCTGACTTCC	CGTGTCTATGTTCTGCGTGTAG
Mmu-Sirt3	GAGCGGCCTCTACAGCAAC	GGAAGTAGTGAGTGACATTGGG
Mmu-Sirt4	GATTGACTTTCAGGCCGACAA	GCGGCACAAATAACCCCGA
Mmu-Sirt5	CCAGTTGTGTTGTAGACGAAAGC	TTCCGAAAGTCTGCCATATTTGA
Mmu-Sirt6	CTCCAGCGTGGTTTTCCACA	GCCCATGCGTTCTAGCTGA
Mmu-Sirt7	GCACTTGGTTGTCTACACGG	TGTCCATACTCCATTAGGACCC
Hsa-Col2a1	TGGACGATCAGGCGAAACC	GCTGCGGATGCTCTCAATCT
Hsa-Acan	ACTCTGGGTTTTCGTGACTCT	ACACTCAGCGAGTTGTCATGG
Hsa-Mmp13	ACTGAGAGGCTCCGAGAAATG	GAACCCCGCATCTTGGCTT
Hsa-Adamts5	GAACATCGACCAACTCTACTCCG	CAATGCCCACCGAACCATCT
Hsa-Gapdh	GGAGCGAGATCCCTCCAAAAT	GGCTGTTGTCATACTTCTCATGG
Hsa-Sirt1	TAGCCTTGTCAGATAAGGAAGGA	ACAGCTTCACAGTCAACTTTGT
Hsa-Sirt2	TGCGGAACTTATTCTCCCAGA	GAGAGCGAAAGTCGGGGAT
Hsa-Sirt3	ACCCAGTGGCATTCCAGAC	GGCTTGGGGTTGTGAAAGAAG
Hsa-Sirt4	GCTTTGCGTTGACTTTCAGGT	CCAATGGAGGCTTTCGAGCA
Hsa-Sirt5	GCCATAGCCGAGTGTGAGAC	CAACTCCACAAGAGGTACATCG
Hsa-Sirt6	CCCACGGAGTCTGGACCAT	CTCTGCCAGTTTGTCCCTG
Hsa-Sirt7	GACCTGGTAACGGAGCTGC	CGACCAAGTATTTGGCGTTCC
